# Reducing stress and alcohol-related behaviors by targeting D1-CRHR1 receptor interactions in the amygdala

**DOI:** 10.3389/fphar.2025.1677510

**Published:** 2025-10-16

**Authors:** Laura Broccoli, Rick E. Bernardi, Francesco Giannone, Anika Hoppe, Kay Jungling, Dasiel O. Borroto-Escuela, Roberto Rimondini-Giorgini, Miguel Pérez de la Mora, Oliver von Bohlen und Halbach, Kjell Fuxe, Hans-Christian Pape, Jan M. Deussing, Rainer Spanagel, Wolfgang H. Sommer, Anita C. Hansson

**Affiliations:** ^1^ Institute of Psychopharmacology, Central Institute of Mental Health, Medical Faculty Mannheim, University of Heidelberg, Mannheim, Germany; ^2^ Institute of Physiology I (Neurophysiology), University of Münster, Münster, Germany; ^3^ Department of Neuroscience, Biomedicum, Karolinska Institutet, Stockholm, Sweden; ^4^ Medical and Surgical Sciences Department (DIMEC), University of Bologna, Bologna, Italy; ^5^ Division of Neuroscience, Instituto de Fisiología Celular, Universidad Nacional Autónoma de México, Mexico City, Mexico; ^6^ Institute for Anatomy and Cell Biology, Universitätsmedizin Greifswald, Greifswald, Germany; ^7^ Molecular Neurogenetics, Max Planck Institute of Psychiatry, Munich, Germany

**Keywords:** intercalated amygdalar cells, corticotropin releasing factor 1 receptor, dopamine D1 receptor (D1R), alcohol use disorder (AUD), amygdala, stress and anxiety, receptor-receptor interactions, genetically modified mice

## Abstract

**Background:**

Basic neuroscience has identified dopamine and corticotropin-releasing hormone (CRH) systems and amygdala circuits as key contributors to drug and alcohol reward, craving, and relapse. However, directly targeting these systems has had no impact on the treatment of addictive disorders. CRH receptor 1 (CRHR1) and dopamine receptor D1 are both modulators of amygdala function. The intercalating cell masses (ITC) regulate intra-amygdala signal flow and are highly enriched in CRHR1 and D1. To date, interactions between these systems have not been extensively examined. We tested these interactions using a combination of *in vivo* pharmacology, genetic targeting and behavioral studies in rodents.

**Methods:**

The impact of CRHR1 activation on D1 was demonstrated using i. c.v. injection of either CRH (2 μg/μL) or stressin I (4 μg/2 μL) in naïve rats followed by D1 receptor autoradiography. We then injected stressin I (0.01 μg/0.5 μL) and stressin I in combination with the D1 antagonist SCH23390 (120 ng/0.5 μL) site-specifically into the ITC and tested animals in the Elevated-Plus-Maze (EPM), followed by saturated D1 receptor autoradiography. Alcohol dependence was also induced in rats and D1^Cre^-Crhr1^−/−^ knockout mice *via* cyclic intermittent alcohol vapor exposure. Following abstinence, rats were used for D1 expression analysis (*in situ* hybridization, D1 autoradiography) and assessment of morphological changes using Golgi-impregnation. In addition, abstinent D1^Cre^-Crhr1^−/−^ mice were analyzed for alcohol drinking and stress-related alcohol drinking (Two-Bottle-Free-Choice, Forced Swim Stress-induced drinking). Double-immunofluorescence immunostainings were performed for D1 and CRHR1 in the amygdala of *Crhr1*-GFP reporter mice.

**Results:**

Pharmacological activation of CRHR1 by i. c.v. injection of CRH or stressin I increased D1 binding sites exclusively in the amygdala, but not in extra-amygdala brain regions. Site-specific CRHR1 activation in the amygdala/ITC was associated with increased anxiety-like behavior that was prevented by co-treatment with SCH23390. In dependent D1^Cre^-Crhr1^−/−^ mice, the D1-CRHR1 interaction appears to be critically involved in maladaptive stress coping and increased relapse propensity. Our immunohistochemical findings also suggest the D1-CRHR1 interaction is dependent on co-localized receptors.

**Conclusion:**

Our findings suggest D1-CRHR1 interactions within the ITC of the amygdala in response to stress, alcohol behavior, and the development of dependence, thereby providing a novel mechanism that may be targetable by therapeutic polypharmacological interference.

## Introduction

Harmful alcohol use accounts for about 5% of the global burden of disease and about 6% of all deaths worldwide (GBD 2016 Alcohol and Drug Use Collaborators, 2018; WHO, 2018). By many measures, the harm caused by alcohol exceeds that of all illicit drugs (Nutt et al., 2010). After prolonged, repeated exposure, alcohol acts as an addictive substance that alters numerous neuromolecular targets and signaling cascades, affecting major neurotransmitter systems such as GABA and glutamate, as well as neuromodulatory systems including dopamine and stress-related peptides, ultimately contributing to the development of alcohol use disorder (AUD) (Lovinger and Roberto, 2023). Development of the clinical condition takes many years and is contingent upon repeated and prolonged periods of brain exposure to intoxicating levels of alcohol. During this process, stress circuits become progressively recruited and contribute to a chronic negative affective state that is likely critical for the transition to compulsive alcohol use (Koob and Schulkin, 2019; Heilig et al., 2010).

Previous studies in rodents with experimentally-induced alcohol dependence have demonstrated a recruitment of the corticotropin releasing hormone (CRH) system in the amygdala. Specifically, an upregulation of CRH receptor type 1 (CRHR1) within the amygdala complex has been linked to an increased behavioral sensitivity to stress, alcohol intake, and seeking behavior (Sommer et al., 2008; Hansson et al., 2006b; Broccoli et al., 2018). This constitutes a key mechanism in the development of a negative emotional state that leads to compulsive alcohol intake. Importantly, in animal models of AUD, blockade of CRHR1 has consistently shown efficacy in reducing anxiety, alcohol consumption and relapse behavior (Sommer et al., 2008; Gehlert et al., 2007; Simms et al., 2014; Cottone et al., 2009; Funk et al., 2006), establishing the progressive sensitization of the amygdala CRH system during the development of dependence as one of the most robust finding in animal models of addiction (Heilig et al., 2011). However, despite the large preclinical evidence, clinical studies with CRHR1 antagonists in AUD and other psychiatric conditions have failed (Kwako et al., 2015; Schwandt et al., 2016), questioning the translatability of targeting CRH systems in disease (Shaham and de Wit, 2016; Murrough and Charney, 2017).

The complexity of the CRH system was first highlighted by the counterintuitive finding that CRHR1 knockout mice exhibited increased, rather than decreased, alcohol intake (Sillaber et al., 2002). This discrepancy was later resolved by Molander et al. (2012), who demonstrated that brain-specific CRHR1 knockout mice displayed the expected decrease in stress-induced alcohol intake. Further proof of the complexity of the CRH system was provided by Refojo et al. (2011) demonstrating that the knockout of CRHR1 in DAergic neurons increased anxiety-like behavior and reduced DA release in the prefrontal cortex (PFC). CRH release from GABAergic neurons of the extended amygdala acts anxiolytically by positively modulating DA release (Dedic et al., 2018). Furthermore, differences in CRHR1 expression levels in response to stress mediate subsequent behavioral responses. For example, differences in the effect of acute stress on motivated behavior in rats have previously been attributed to expression levels of CRHR1 in DAergic neurons in the ventral tegmental area (VTA) (Zalachoras et al., 2022). Studies in brain slices have shown increased activity of midbrain DA neurons upon CRH administration, providing further evidence of a functional CRH-DA interaction (Hahn et al., 2009; Wanat et al., 2008; Riegel and Williams, 2008). Similarly, DA can activate CRH signaling *via* DA receptors through local neuronal networks in the bed nucleus of the stria terminalis (BNST) (Meloni et al., 2006; Orozco-Cabal et al., 2008; Kash et al., 2008). These examples suggest regional and neuronal population-specific interactions between DA and CRH systems.

Extensive evidence highlights the crucial role of synaptic transmission within the central nucleus of the amygdala (CeA) in governing alcohol-related behaviors and the neuroadaptive processes associated with alcohol dependence. Acute alcohol enhances GABAergic transmission in the CeA through both pre- and postsynaptic mechanisms, while chronic alcohol exposure leads to an overall increase in baseline GABA transmission. In contrast, acute alcohol suppresses glutamatergic signaling *via* effects on NMDA and AMPA receptors, whereas prolonged alcohol exposure results in upregulation of NMDA receptor-mediated transmission. CRH influences alcohol-related behaviors and modulate alcohol’s impact on CeA neurotransmission (Roberto et al., 2021). In addition, the amygdala is a key center of emotional regulation and orchestrates adaptive responses to stressful stimuli (LeDoux, 2000). Within this highly heterogeneous structure, the basolateral amygdala (BLA, consisting mainly of glutamatergic neurons), receives information about the external environment that is relayed *via* glutamatergic projections to the neighboring CeA (consisting mainly of GABAergic interneurons), the main output region (McDonald, 1982). The BLA is surrounded by distinct inhibitory clusters of intercalated neurons (ITCs), densely packed GABAergic neurons that can be distinguished from neighboring neurons by their electrophysiological and molecular properties (Nitecka and Ben-Ari, 1987; McDonald and Augustine, 1993; Pare and Smith, 1993; Marcellino et al., 2012; Collins and Pare, 1999; Millhouse, 1986), in particular a very high density of D1 receptors (Fuxe et al., 2003; Jacobsen et al., 2006; Asede et al., 2022) and CRHR1 (Justice et al., 2008). The medial ITC clusters at the BLA-CeA junction receive input from the BLA and modulate CeA activity *via* feed-forward inhibition microcircuits (McDonald, 1985; Royer et al., 1999; Duvarci and Pare, 2014) in a manner that is relevant for stress and fear responses and anxiety (Busti et al., 2011; Hagihara et al., 2021; Amano et al., 2010). Interestingly, DA hyperpolarizes ITCs through D1 receptors and substantially suppresses their excitability, resulting in a disinhibition of the BLA and CeA (Marowsky et al., 2005) and an anxiogenic response (de la Mora et al., 2005). Thus, the activation of D1 in ITC cells appears to be essential for the disinhibition of the CeA, likely caused by silencing of the ITC network.

The reviewed literature suggests that both DA and CRH systems are modulators of amygdala function and play important roles in the control this region exerts on emotional regulation. So far, interactions between these systems have not been extensively examined. In this study we tested a functional D1-CRHR1 interaction using a combination of *in vivo* pharmacology, genetic targeting, behavioral studies linked to D1 availability. Further mechanistic investigations were undertaken using whole-cell patch-clamp recordings and Bioluminescence Resonance Energy Transfer (BRET) experiments. Our results suggest that D1-CRHR1 interactions occur in the amygdala in response to stress and the development of alcohol dependence, thereby providing a novel mechanism to treat anxiety-related disorders and relapse to drug-seeking behavior.

## Materials and methods

### Animals and experimental design

Male Wistar rats (Charles Rivers, Germany) were used to establish a D1-CRHR1 interaction using intracranial pharmacological treatments and the measurement of D1 expression levels in alcohol dependence. Male D1^Cre^-Crhr1^−/−^ knockout mice (C57Bl/6 genetic background (Bernardi et al., 2017)) were used to establish a D1-CRHR1 interaction using intracranial pharmacological treatments, the characterization of anxiety-related behaviors, and the measurement of D1 expression levels in alcohol dependence. Co-localization between D1 and CRHR1 was assessed within the amygdala nuclei using double immunohistochemistry assays performed on brain sections of *Crhr1*-GFP reporter male mice (C57Bl/6 genetic background (Justice et al., 2008)). In order to functionally characterize the receptor-receptor interactions in the amygdala, electrophysiological studies (whole-cell patch-clamp recordings) were performed on intercalated cell cluster of *GAD67*-GFP male mice (C57Bl/6 genetic background (Tamamaki et al., 2003)). To further investigate the molecular mechanism underlying D1-CRHR1 cross-talk, BRET assays were carried out in HEK293 cells.

Food and water were available *ad libitum.* Holding rooms for all animals were kept under controlled conditions of light (12 h light-dark cycles from 07:00 to 19:00), temperature (20 °C–22 °C) and humidity (65%). Wistar rats, *Crhr1*-GFP and *GAD67*-GFP mice were group-housed, while D1^Cre^-Crhr1^−/−^ mice and their littermate controls were single-housed. All behavior experiments were conducted in the dark cycle.

### Intracranial surgeries and treatments

Guide cannulas (Plastic One, Roanoke, VA) were first implanted. The exact position of the cannula was calculated by Bregma identification (rat brain atlas (Paxinos and Watson, 1998); mouse brain atlas (Paxinos and Franklin, 2007)). Rats and mice were single-housed for 7 days in order to recover completely from the surgery. After recovery, rats and mice were administered intracerebroventricular (i.c.v.) microinjections for D1 receptor autoradiography, and in rats, bilateral amygdala microinjections for Elevated Plus Maze (EPM) testing + D1 receptor autoradiography.

In rats, the coordinates for i. c.v. microinjections of artificial cerebrospinal fluid (aCSF, 2 µL, Sigma-Aldrich, Missouri, United States), CRH (2 µg/2 µL, CRHR1 agonist, Ki = 0.95 nM (Donaldson et al., 1996), Tocris Bioscience, Bristol, United Kingdom), stressin I (4 µg/2 µL, CRHR1 agonist, Ki = 1.5 nM (Rivier et al., 2002), Tocris Bioscience, Bristol, United Kingdom) and SCH23390 (D1 high-affinity antagonist, Ki = 0.3 nM (Bourne, 2001), Tocris Bioscience, Bristol, United Kingdom) were: Bregma posterior −0.80 mm, lateral ±1.40 mm and ventral −3.2 mm. Intra-amygdala injected Wistar rats were bilaterally infused with aCSF (0.5 µL/hemisphere) or stressin I (0.01 µg/hemisphere) or stressin I with SCH23390 (120ng/hemisphere). The compounds were injected in a total volume of 0.5 µL per hemisphere. The oordinates for intra-amygdala injections in rats were: Bregma posterior −1.80 mm, lateral ±4.2 mm and ventral −7.9 mm. All compounds were injected with a speed rate of 250 nL/min using a micro-infusion pump (Harvard Apparatus, Massachusetts, United States) and Hamilton syringe (25 µL) with a microinjector (Plastics One, Roanoke, VA) protruding 0.5 mm below the implanted guide cannula to reach the target area. The 28 g needle was kept in position for 1 minute after the end of the injection to avoid back-flow.

D1^Cre^-Crhr1^−/−^ and Crhr1^f/f^ littermate controls received i. c.v. injections of aCSF (2 µL injection volume) or stressin I (0.5 µg/2 µL), according to the coordinates: Bregma posterior −0.10 mm, lateral +/-0.85 mm and ventral −2.0 mm. All compounds were injected as described above for rats, with the 33 g needle kept in position for 1 minute after the end of the injection to avoid back-flow.

All animals were sacrificed by decapitation 1 h after microinjection. Brains were quickly removed, frozen in −40 °C isopentane and kept at −80 °C until use. D1 receptor autoradiography was performed on brain sections. Animals with incorrect cannula placement were excluded from the analysis.

### Behavioral measures in rats

#### Elevated-Plus-Maze (EPM)

The EPM apparatus consisted of two open arms (50 cm × 10 cm) crossed at right angles with two enclosed arms of the same length. The entire apparatus was elevated 50 cm above the floor. The light intensity in the apparatus was set to 30 lux. Rats were acclimated to the room for 30 min, immediately following intracranial injections as described above. Subsequently, each animal was placed in the center of the maze, facing a closed arm, and left free to explore in all four directions for 5 min. The percentage of time spent in the open arms and number of entries into the open arms were used as measures of anxiety-like behavior. All arms were cleaned with 50% ethanol solution and dried after each trial.

### Behavioral measures in D1^Cre^-Crhr1^−/−^ mice

#### Locomotor activity

Homecage locomotion was measured in D1^Cre^-Crhr1^−/−^ mice and Crhr1^f/f^ littermate controls using a passive infrared sensor incorporated in the Mouse-E-motion Universal Data Logger (Infra-E-Motion GmbH, Hamburg, Germany). Warmth radiation emitted by the animal was recorded by the sensor. Since plastic cages were impermeable for infrared radiation, external inputs from outside or neighbor cages were not detected. All movements were recorded every 4 min for 3 consecutive days and were expressed as activity/4 h.

Locomotor activity in the Open Field was measured for 15 min in 8 TruScan activity arenas (Coulbourn Instruments, United States), similar to assessments described previously (Bernardi et al., 2014; Bernardi et al., 2017). Each monitor consists of a clear acrylic plastic test cage (27 × 27 × 39 cm) placed inside a monitoring unit that records *via* computer ambulatory beam interruptions from infrared photocell emitter/detector pairs evenly spaced along each axis. Light intensity was set up at 20 lux. Prior to testing, D1^Cre^-Crhr1^−/−^ mice and Crhr1^f/f^ littermate controls were acclimated to the room for 30 min. Subsequently, animals were placed in the Truscan chamber for the 15-min assessment. Total locomotor activity was measured as distance travelled (cm) and the time spent in the center, expressed in seconds (s), was used as an index for anxiety-like behavior.

### Dark-light box

The Dark-Light box consists of a dark, protected compartment connected by a short tunnel to a bright (40 lux) arena. Prior to testing, D1^Cre^-Crhr1^−/−^ mice and Crhr1^f/f^ littermate controls were acclimated to the room for 30 min. Subsequently, an animal was placed in the dark compartment for a 30-s habituation period, after which the door between the dark and light compartments was removed, and an animal was allowed to move freely in both arenas for 5 min. The time spent in the light compartment was considered as a measure of anxiety. The apparatus was cleaned with 50% ethanol solution and dried after each trial.

### Fear conditioning

The conditioning apparatus consisted of two different chambers inserted in sound- and light-protected isolation cubicles (Habitest H10-24TA, Coulbourn Instruments, United States). Context A (17 cm × 18 cm × 32 cm) had two transparent walls and stainless steel grid floors (H10-11M-TC, Coulbourn Instruments, United States) from which 0.6 mA scrambled footshock was delivered from a precision animal shocker (H13-15, Coulbourn Instruments, United States). Context B had four transparent walls, stainless steel grid floors, and was 30 cm × 25 cm × 25 cm in size (Med Associates, United States). The sound stimulus was a 5,000-Hz, 80- to 85-dB tone and delivered *via* speakers into the chamber. The movements of the tested animal were recorded with a digital video camera mounted at the ceiling of the cubicle and analyzed for the percentage of freezing using FreezeView software (Actimetrics Software) (Waltereit et al., 2008). Prior to each session, D1^Cre^-Crhr1^−/−^ mice and Crhr1^f/f^ littermate controls were acclimated to the room for 30 min. During the acquisition phase, mice were placed in context A. After a 3-min habituation phase, animals were presented with five exposures to a 30-s acoustic stimulus that terminated with a single foot shock. The five stimulus/shock pairings occurred at random intervals. On the next day contextual fear was assessed by placing the mice in context A without delivery of the tone or foot shock. Freezing was analyzed for 6 min. Mice were then moved back into their home cages for at least 5 h before testing them for cued fear. Auditory cued fear was assessed by placing the mice in context B. After 3 min of acclimatization, the sound stimulus was presented for 6 min but without foot shocks. Freezing behavior was recorded during the session.

### Two-bottle free-choice procedure

Voluntary alcohol intake was measured in D1^Cre^-Crhr1^−/−^ and Crhr1f^/f^ littermate controls during chronic intermittent alcohol experiments described below using a two-bottle free-choice paradigm, according to standardized procedures (Spanagel and Holter, 1999). During the initial phase of the experiment, mice were habituated to alcohol taste by substituting one of the two tap water bottles with an ethanol solution. Alcohol concentration was increased every 3 days (2%, 4%, 8%, v/v) up to 12% ethanol for the remainder of the experiment. The position of the bottles was switched randomly to avoid a location preference. All bottles were weighed and freshly prepared every 3 days. Mice were weighed once a week. The amount of alcohol consumption is expressed as the absolute amount of solution (taking into account alcohol density (0.8 g/mL)) consumed each day with respect to the weekly weight of the animal (g/kg/day). Baseline drinking was defined as the level of stable alcohol intake maintained for three consecutive measurements.

### Forced swim stress (FSS)

Repeated FSS was performed in alcohol-dependent D1^Cre^-Crhr1^−/−^ mice and Crhr1^f/f^ littermate controls. Prior to each FSS session, animals were acclimated to the room for 30 min. Mice were placed in a glass cylinder (25 cm high, 14 cm wide) filled two-thirds with water to avoid the ability of mice to touch the bottom with the tail. The water temperature was maintained at 21 °C. Each trial lasted for 5 min and mice were tested for 3 consecutive days. After each trial, mice were gently dried and moved back to their home cage with free access to water and alcohol. On the following 3 days alcohol bottles were weighed daily to observe any variation in alcohol intake.

### Induction of alcohol dependence by cyclic intermittent alcohol vapor exposure (CIE) in rats and mice

Rats and mice were exposed to daily cycles of intermittent alcohol vapor intoxication and withdrawal, a paradigm that allows a high degree of control over brain alcohol levels and induces behavioral and molecular changes relevant for the pathophysiology of alcoholism (Meinhardt and Sommer, 2015; Rimondini et al., 2002; Becker and Lopez, 2004; Eisenhardt et al., 2015).

### CIE in rats

Ethanol vapor exposure in rats was performed in custom made chambers (85 × 85 × 67 cm) designed to accommodate four Type-IV cages (up to 16 rats per chamber). Alcohol (96%) was delivered by dosing pumps (Knauer, Berlin, Germany) into electrically heated stainless-steel coils (60 °C), combined with an airflow of 18 L/min, and subsequently released into the chambers. Rats were first allowed to habituate to the chambers for 1 week. For the next 7 weeks, rats were exposed to daily 16 h of continuous exposure to alcohol (Sigma-Aldrich, Missouri, United States), followed by 8 h of withdrawal.

### CIE in mice

Alcohol vapor exposure was performed using a chamber system (La Jolla Alcohol Research, La Jolla, CA, United States). A peristaltic Q-pump (Knauer, Berlin, Germany) delivered 96% ethanol into a heated round-bottom flask (0.44 mL/min), where it was vaporized and carried by an airstream (5.9 L/min) into four individual chambers *via* side-arm tubing. Each chamber was connected to a vacuum system to ensure constant circulation and maintain ethanol concentrations of 10–15 mg/L air. D1^Cre^-Crhr1^−/−^ mice and Crhr1^f/f^ controls were habituated to the chambers for 1 week. CIE in mice lasted 4 weeks, with each cycle of 16 h continuous exposure to alcohol and 8 h of withdrawal occurring five consecutive days per week. Before each cycle of exposure mice were i. p. injected with alcohol (1.6 g/kg; 8% w/v; 96% EtOH) and blood alcohol concentration (BAC) was stabilized by administration of 1 mmol/kg of pyrazole (Sigma-Aldrich, Missouri, United States), an alcohol dehydrogenase inhibitor. Control mice were handled similarly, but received injections of saline and pyrazole (Becker and Lopez, 2004; Lopez and Becker, 2005; Griffin et al., 2009).

### BAC measurements

Blood (∼20 µL) was sampled from the lateral tail vein of exposed rats and mice for BAC measurements two times per week. BACs were determined using an AM1 Analox system (Analox Instruments Ltd., London, United Kingdom, BAC range: 150–300 mg/dL per cycle). Following intermittent exposure to alcohol animals were subjected for several weeks of abstinence.

### Alcohol withdrawal severity scores in mice

After the last intoxication cycle, alcohol withdrawal severity was assessed (Mutschler et al., 2010; Uhrig et al., 2017) in D1^Cre^-Crhr1^−/−^ mice and Crhr1^f/f^ controls. Animals were scored for withdrawal symptoms immediately after the last vapor exposure cycle (time 0), and during the next 4, 8 and 12 h. Neuro-vegetative withdrawal signs like tremor, piloerection, tail rigidity, vocalizations, teeth chattering and wet dog shakes (WDS) were assessed by observing each mouse for 5 min. Each symptom was scored as 0, 1 and 2, indicating low, middle and high severity respectively. The sum of the incidence of each sign represents the entire withdrawal score. Withdrawal scores were assessed in both dependent and control mice, starting from the end of the cycle of ethanol intoxication (time 0 h) and the measurements were repeated after 4 h, 8 h and 12 h.

### Saturated receptor autoradiography

The D1 antagonist [^3^H]-SCH23390 (specific activity = 80.5 Ci/mmol; Kd = 0.7nM, B_max_ = 347.0 ((Schulz et al., 1985), Perkin-Elmer, Massachusetts, United States)) was used as the radio-labeled ligand. SKF38393 (*Kd* = 9.9 nM (Dubois et al., 1986), Tocris Bioscience, Bristol, United Kingdom), a D1 selective partial agonist, was the cold competitor to identify non-specific binding (Hirth et al., 2016; Bernardi et al., 2015; Sommer et al., 2014). First, 12 µm coronal brain sections were dipped in the pre-incubation buffer containing 50 mM Tris (pH 7.4), 5mM MgCl2, 1 mM EDTA at room temperature for 15 min. This step was repeated a second time with fresh buffer. Then, the incubation buffer (10 nM [^3^H]-SCH23390, 50 mM Tris (pH7.4), 5 mM MgCl_2_, 1 mM EDTA, 100 mM NaCl, 1 mM DTT, 0.1% bovine serum albumin-BSA) was applied on each slide and kept at 30 °C for 2 h. Non-specific binding was estimated by incubating adjacent sections in a buffer containing a mix of 10 nM [^3^H]-SCH23390 and 1 µM SKF38393. Sections were washed twice for 2 min in cold 50 mM Tris-HCl buffer (pH 7.4) and for 2 min in ice cold distilled water before being dried under a stream of cold air.

### *In situ* hybridization

*In situ* hybridization was performed as previously described by Hansson et al. (2006a) and Sommer et al. (2008). 12 μm brain sections were incubated in 4% paraformaldehyde in PBS for 15 min, washed for 10 min in PBS, and twice in sterile water for 5 min. After treatment with 0.1M HCl solution for 10 min and two times with PBS for 5 min, sections were incubated in 0.1M triethanolamine (pH8) and 0.25% acetic anhydride buffer for 20 min. Subsequently, sections were washed twice in PBS for 5 min, once in sterile water for 1 min and dehydrated in a graded series of ethanol (70%, 80%, 99%) for 2 min. After air drying, sections were stored at −80 °C.

A D1 rat-specific riboprobe was generated from rat cDNA (RefSeq: NM_012546.2, position from 60bp to 1400bp). Antisense and sense RNA probes were obtained by incubating 200 ng DNA with transcription buffer (Ambion^®^ Applied Biosystems, Darmstadt, Germany with 12.5 nmol of ATP, CTP, GTP, 50 pmol UTP and 125 pmol [^35^S]-UTP [1,250 Ci/mmol, Perkin Elmer, Rodgau, Germany, 1U RNase inhibitor and 1U RNA polymerase (Roche Molecular Biochemicals, Mannheim, Germany)] at 37 °C for 90 min. Afterwards, the DNA was digested with RNase-free DNase (Roche Molecular Biochemicals, Mannheim, Germany) at 37 °C for 20 min and the transcripts were purified using spin columns (illustra™ Microspin™ S-200 HR Colums, GE Healthcare, Munich, Germany). Sections were first incubated in a pre-hybridization buffer (50% deionized formamide, 50 mM Tris-HCl pH7.6, 25 mM EDTA pH8.0, 20 mM NaCl, 0.25 mg/mL yeast tRNA, 2.5 × Denhardt’s solution (Invitrogen, Darmstadt, Germany) at 37 °C for 2–4 h. Then, the sections were hybridized with 100 µL hybridization buffer (50% deionized formamide, 20 mM Tris-HCl pH7.6, 10× Denhardt’s solution, 5 mg/mL yeast tRNA, 1 mg/mL polyadenylic acid, 10 mM EDTA pH8.0, 150 mM DTT, 330 mM NaCl, 10% dextransulphate) added with 1 × 10^6^ cpm of either labeled antisense RNA or sense RNA and immediately covered with siliconized coverslips. After overnight incubation in a humidified chamber at 55 °C, coverslips were removed with 3 consecutive washing steps using 1x standard saline citrate (SSC) solution at 42 °C for 40 min and the sections were washed in 0.5× SSC/50% formamide for 1 h at 42 °C. Following two additional washing steps in 1× SSC for 30 min at 42 °C the sections were treated with 1 μg/mL RNaseA in RNase buffer (0.5 M NaCl, 10 mM Tris pH8.0, 1 mM EDTA pH7.5) for 1 h at 37 °C. After two washing steps in 1× SSC for 30 min at 55 °C followed by a brief washing in 1×SSC at RT, the sections were dehydrated in graded ethanol and air-dried. Sections that were damaged or contained artifacts within the regions of interest were excluded from the analysis.

### Densitometric measurements

Fuji Imaging Plate BAS-TR2025 (GE Healthcare Life Science, Pittsburgh, United States) and BAS-TR were exposed to brain sections for D1 autoradiography and *in situ* hybridization. After 1 week, Fuji plates were scanned with the phosphorimager (Typhoon FLA 700, GE Healthcare, Germany). The digital images obtained, were used to measure the signal density by MCID Image Analysis Software (Imaging Research Inc., United Kingdom). Signal density was measured as minimal detectable change (MDC) units per mm^2^. For receptor autoradiography assays, the measurements were converted into fmol of receptor per mg protein tissue by plotting values in a standard curve obtained by a [^3^H]-microscales (Amersham, GE Healthcare Life Sciences, Pittsburg, United States). Binding in femtomoles per milligram (fmol/mg) was calculated based on the specific activity of the radioligand. For *in situ* hybridization assays the values were compared against standard curves generated using [^14^C]-Microscales (Amersham, GE Healthcare Life Sciences, Pittsburgh, United States).

### Double fluorescence immunohistochemistry

Male *Crhr1*-GFP mice were deeply anesthetized with isoflurane and intracardially perfused with 0.9% NaCl solution with 10,000 IE/1 heparine and then with fixative solution (phosphate buffer, containing 4% paraformaldehyde and 14% saturated picric acid). Brains were collected, post-fixed for 1h, dehydrated in 1xPBS solution added with 10% sucrose for 3 days and finally frozen at −80 °C. Fluorescent double-labeling immunohistochemistry were performed as described by Bernardi et al. (2017). 12 µm coronal amygdala sections were mounted on gelatin-coated slides and stored in −20 °C until the use. Sections were brought to room temperature, rehydrated, rinsed in 0.01 M PBS buffer, and incubated with a mixture of two primary antibodies: rat anti-D1 (Sigma-Aldrich, Missouri, United States, 1:400) and rabbit anti-GFP (ThermoFisher Scientific, United States, 1:300) at 4 °C overnight. The sections were rinsed in 0.01M PBS/0.3% Triton buffer, and a mixture of the two secondary antibodies (conjugated with Alexa 488 or Alexa 555: AlexaFluor 488-labeled donkey anti-rabbit, 1:200, and AlexaFluor 555-labeled donkey anti-rat, 1:100) were added and incubated for 1–2 h at room temperature. Sections were rinsed briefly and mounted in mounting medium (Dako, Carpinteria, CA). Confocal images were collected on a Leica TCS SP5 (DM IRE2 stand) with 20 × /0.70 NA and 63 × /1.40 NA oil objectives; all quantitative analyses used the ×20 stacks. Sequential frame acquisition employed 25 %-power 488 nm and 561 nm lines, with emission windows of 500–561 nm (green) and 599–670 nm (red) detected on PMTs set to 950 V gain (offsets −2.5 V and −1 V, respectively). Z-stacks (1,024 × 1,024 pixels; 0.76 µm × 0.76 µm in XY; 24 slices at 0.49 µm) were recorded with 2× line averaging at 400 Hz and saved as 8-bit TIFFs.

### Golgi-impregnation

For measurements of structural changes for each group, rat brains from control and alcohol-dependent rats were investigated. Brains were divided in the hemisphere by a longitudinal cut. A complete hemisphere was processed for silver impregnation. The impregnations were performed according to the Golgi-Cox procedure using Rapid GolgiStain reagents kit and the protocol provided by the supplier (FD NeuroTechnologies, United States). After successful impregnation the brain was sectioned into 120 µm thick slices by using a Leica VT 1000S vibratome (Leica, Germany). These thick sections were mounted on gelatine-coated slides and coverslipped with Merckoglas (Merck, Germany). Quantitative three-dimensional analyses of dendritic fragments and their dendritic spines were conducted as described previously (von Bohlen und Halbach et al., 2006) using a combined hardware-software system (NeuroLucida, version 9.12, MBF Bioscience, United States) controlling the x-y-z axis of the microscope (Axioscop Imaging, Zeiss, Germany) and a microscope-mounted digital camera (AxioCam HRc; Zeiss, Germany). Since the size of the dendritic spines is near the Abbe limit, a specific oil objective with a high numerical aperture (NA) was used for the three-dimensional reconstruction (×100 oil plan-apochromate objective (NA: 1.4; Zeiss, Germany)). Using the combined hardware-software system, z-stacks (step size 0.125 µm) were generated and used for reconstruction. Between 18 and 26 individual dendrites were mapped per region and brain. In total, more than 10,000 individual dendritic spines were reconstructed per brain area (17733 in the BLA, 18815 in the CEA, 12431 in the MEA). Spine densities and mean spine length were calculated from the reconstructed dendrites with the help of NeuroExplorer (version 9.12; MBF Bioscience, United States). The n values for the statistical analysis were based on animal numbers (n) and not on numbers of analyzed elements (n_e_).

### Electrophysiological experiments

Naïve *GAD67*-GFP mice were anesthetized and sacrificed by decapitation. Amygdala coronal sections (300 µm thickness) from *GAD67*-GFP mice were cut with the vibratome and whole-cell patch-clamp recordings were performed on ITCs as described previously (Jungling et al., 2008). Briefly, GFP-expressing ITCs were identified by fluorescent microscopy and the recording was carried out either in the current-clamp mode to analyze DA-dependent effects on the membrane potential or stressin I-induced currents, respectively, using an EPC-10 patch clamp amplifier (HEKA). The membrane potential was set between −60 and −65 mV by manual positive current injections. For whole cell recordings a potassium gluconate-based intracellular solution was used (in mM: 10 NaCl, 88 K-gluconate, 20 K_3_-citrate, 10 HEPES, 3 BAPTA, 1 MgCl_2_, 0.5 CaCl_2_, 15 phosphocreatine, 3 Mg-ATP, 0.5 Na-GTP; pH: 7.25; 290–300 mOsmol) and the pipette resistance was about 3 MΩ. All recordings were done in aCSF at 32 °C. Dopamine (20 μM; Sigma-Aldrich, Missouri, United States) was applied by fast bath perfusion (2.5–3 mL/min) together with GluR antagonists (10 µM DNQX and 25 µM DL-AP5, Abcam) and GABA-R antagonists (25 µM Gabazine and 2.5 µM CGP55845, Abcam). To investigate CRHR1-dependent effects on D1-signaling, a subset of slices was pretreated with the CRHR1-specific agonist stressin I (250 nM; Sigma-Aldrich, Missouri, United States) 45 min prior to recordings. DA-induced changes of the membrane potential were analyzed and compared with the stressin I pre-treatment effect.

### Bioluminescence resonance energy transfer (BRET) assay

Human DA D1 and CRHR1 coding sequences without their stop codons were amplified from 3xHA-D1R-pcDNA and CRHR1-pcMV6 vectors and then sub-cloned into humanized pGFP2-N1 vectors (PerkinElmer, Waltham, MA, United States) and humanized pRluc-N3 vector (Packard Bioscience, Barcelona, Spain) respectively. For transfection, HEK293T cells were plated in 6-well dishes at a concentration of 1× 106 cells/well or in 75 cm^2^ flasks and cultured overnight before transfection. Cells were transiently transfected using TransFectin (Bio-Rad, Sweden). Forty-eight hours after transfection, HEK293T27 cells transiently transfected with constant (1 μg) or increasing amounts (0.12–5 μg) of plasmids encoding for D1-Rluc and CRHR1-GFP2 respectively, were rapidly washed twice in PBS, detached, and re-suspended in the same buffer. Cell suspensions (20 µg protein) were distributed in duplicate into the 96-well microplate black plates with a transparent bottom (Corning 3651) (Corning, Stockholm, Sweden) for fluorescence measurement or white plates with a white bottom (Corning 3600) for BRET determination. For BRET2 measurement, coelenterazine-400a also known as DeepBlue™C substrate (VWR, Sweden) was added at a final concentration of 5 µM. Readings were performed 1 min after using the POLARstar Optima plate-reader (BMG Labtechnologies, Offenburg, Germany) that allows the sequential integration of the signals detected with two filter settings: 410 nm (with 80 nm bandwidth) and 515 nm (with 30 nm bandwidth). The BRET2 ratio is defined as described in (Borroto-Escuela et al., 2013; Borroto-Escuela et al., 2010).

### Statistical analysis

All experimental data are presented as means ± SEM and the chosen criteria of significance for all the analysis was *p < 0.05, **p < 0.01, ***p < 0.001. Statistical analysis was performed by the software Statistica 10 (StatSoft, Tulsa, United States). Graphical representations of the data were created using Prism software (GraphPad, San Diego, United States). Results obtained by receptor autoradiography and *in situ* hybridization assays had homogeneous variance within respective regions and were therefore compared by region-wise one-way analysis of variance (ANOVA), followed by Holm-Bonferroni corrections (Holm, 1979; Gaetano, 2013). Two-way ANOVA analysis was applied to the D1 binding data from pharmacological treatment of D1^Cre^-Crhr1^−/−^ mice, followed by Fisher’s PLSD *post hoc* tests. Locomotor activity (Open Field) and anxiety-like behaviors (Open Field, EPM, Dark-Light box) were analyzed by one-way ANOVA. Homecage locomotion and fear conditioning were assessed by repeated measures ANOVA, followed by Newman-Keuls *post hoc* tests, where indicated. All drinking data (daily alcohol consumption and withdrawal scores) were analyzed using repeated measure ANOVA, followed by *post hoc* Newman–Keuls tests, where indicated. FSS and stress-induced ethanol intake after repeated FSS were analyzed by two-way ANOVA with Fisher´s *post hoc* test. Alcohol consumption in mice was analyzed using two-way ANOVA with repeated measures, followed by Newman-Keuls *post hoc* tests, where indicated. Spine density measurements were conducted using independent samples *t*-tests. For electrophysiological experiments, statistical analysis during drug perfusion was performed for the last 10 min of every condition using two-way ANOVA followed by Bonferroni’s *post hoc* test and comparisons were performed using independent samples *t*-tests. BRET isotherms were fitted using a nonlinear regression equation assuming a single binding site, which provided BRET_max_ and BRET_50_ values. Data obtained from BRET assays were analyzed by one-way ANOVA followed by Tukey’s Multiple Comparison post-test.

## Results

### Activation of CRHR1 increases D1 binding primarily in the amygdala

**FIGURE 1:**
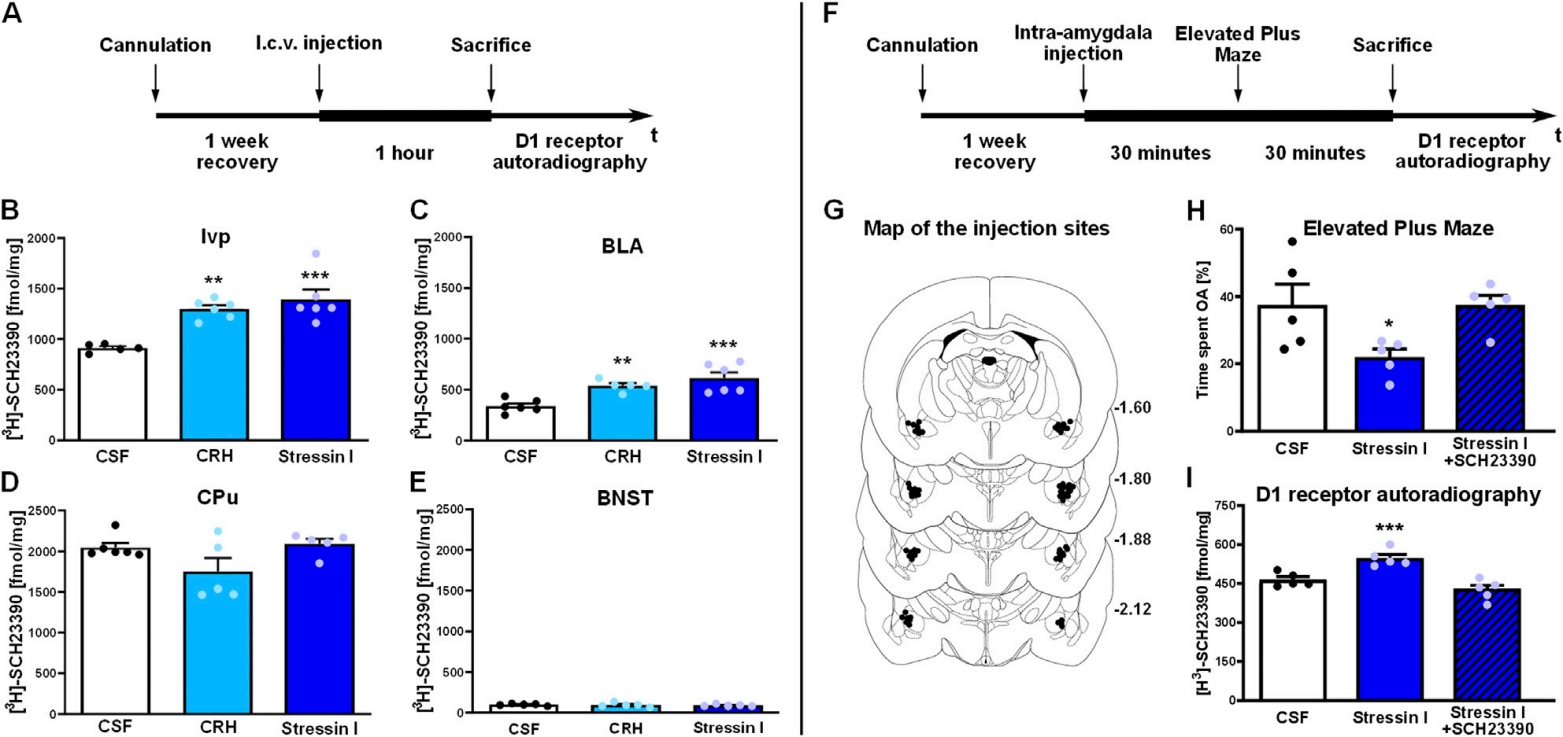
(*Left*, **(A–E)** CRH receptor activation increases D1 receptor binding sites in the amygdala, but not in extra-amygdala regions. **(A)** Time line of the experiment: Rats were bilaterally cannulated close to the lateral ventricle. After 7 days recovery, rats were i.c.v injected with either 2 µL of CSF (white bars), CRH (2 µg in 2 µL injection volume, light blue bars) or the CRHR1-specific agonist stressin I (4 µg in 2 µL injection volume, dark-blue bars). One hour after i.c.v.-injection rats were sacrificed for D1 autoradiography. (*Left*, **(B–E)** Data show D1 binding sites measured by saturated [^3^H]-SCH23390 receptor autoradiography in amygdala regions Ivp **(B)** and BLA **(C)** and in extra-amygdala regions CPu **(D)** and BNST **(E)** Data are shown as individual data points with the mean ± SEM (n = 5–6/group), *p < 0.05, **p < 0.01, ***p < 0.001. BLA, basolateral amygdala; BNST: Bed nucleus of the stria terminalis; CPu: caudate putamen; Ivp: ventral paracapsular island. (*Right*, **(F–I) (F)**. D1 mediates the anxiogenic effects of CRHR1 activation in the amygdala. **(F)** Time line of the experiment. Rats were bilaterally cannulated within the amygdala region (Bregma posterior −1.80 mm, lateral ±4.2 mm and ventral −7.9 mm Paxinos and Watson (1998). After 7–10 days of recovery, rats were injected with either CSF vehicle (0.5 µL per hemisphere, white bars), stressin I (0.01 µg in 0.5 µL per hemisphere, dark-blue bars) or co-injected with both stressin I and D1 antagonist (0.01 µg stressin I+120 ng SCH23390/hemisphere, 0.5 µL/hemisphere, dashed dark-blue bars). One hour after injection animals were sacrificed for receptor autoradiography. **(G)** Schematic illustration showing precision of intra-amygdala injections. **(H and I)** Intra-amygdala injection of stressin I induced anxiety-like behavior as assessed by reduced percentage of time spent in the open arms (OA) during 5 min in the EPM and an increase in D1 binding sites (saturated [^3^H]-SCH23390 receptor autoradiography). Co-treatment with the D1 antagonist SCH23390 blocked both the anxiogenic effect of CRHR1 as well as the increase in D1 receptor binding. Data are shown as individual data points with the mean ± SEM (n = 5/group). *p < 0.05, **p < 0.01, ***p < 0.001.

A cohort of male Wistar rats (n = 18) were equipped with i. c.v cannulae for central administration of CRHR1 ligands. As outlined in Figure 1A, 1 hour after i. c.v. injection of CRH (the natural ligand for both CRHR1 and CRHR2), the CRHR1 agonist stressin I, or aCSF as vehicle, rats were sacrificed and D1 receptor binding was assessed under saturated conditions by autoradiography on brain sections using the D1-specific radio-labelled antagonist [^3^H]**-**SCH23390. Stimulation of CRHR1 *via* CRH or Stressin I strongly upregulated D1 binding sites in almost all amygdala regions, but especially within the ITCs (representative brain regions, ventral paracapsulated island (Ivp) and BLA shown in Figures 1B,C, respectively, mean ± SEM and statistics for all regions are given in Supplementary Tables S1 and S2, respectively). Notably, we found the highest binding levels in the ITCs, in which some of the clusters reached levels nearly as high as those in the striatal regions, while BLA and CeA showed lower D1 binding. The effect obtained by CRH treatment was not as strong as that of stressin I. No effects on D1 binding after CRHR1 activation by either ligand were found outside the amygdala (e.g., caudate putamen, CPu, Figure 1D, and BNST; Figure 1E).

### D1 mediates the anxiogenic effects of CRHR1 in the amygdala

After having shown that brain-wide activation of CRHR1 causes an amygdala-specific increase in D1 binding sites, we wanted to link this effect to local activation of CRHR1 in the amygdala and establish its behavioral and functional consequences. A cohort of rats (n = 15) was equipped bilaterally with cannula guides close to the ITC, at the BLA-CeA junction. As outlined in Figure 1F, after 1 week of recovery, rats received intra-amygdala injections of stressin I (0.01 µg in 0.5 µL per hemisphere) or vehicle (injection sites shown in Figure 1G). As expected from previous studies (Smagin et al., 2001; Bruchas et al., 2009), stressin I produced anxiety-like behavior in the EPM. Stressin I-treated rats showed reduced exploration of the open arm (% time spent in the open arms, F [2.12] = 4.6, p = 0.033, η^2^_p_ = 0.434), but not entries into the open arms (% open arm entries, F [2.12] = 1.5, p > 0.05; *data not shown*). This effect on % time spent in the open arms was fully blocked by co-treatment with the D1 antagonist SCH23390 (Figure 1H). The behavioral specificity of these results was demonstrated by the absence of an effect on general motor behavior. Locomotor activity, as measured by the total number of entries in the closed arms, was not significantly different between the treatment groups (F [2.12] = 3.5, p > 0.05 (mean ± SEM: CSF: 10.6 ± 0.5; Stressin I: 10.2 ± 1.2; Stressin I + SCH23390: 6.8 ± 1.5); *data not shown*). Similar to the i. c.v. injection of stressin I, intra-amygdala injection of the CRHR1 agonist resulted in an increase in D1 binding in all amygdala nuclei and ITCs (Figure 1I). This increase was prevented by co-treatment with the D1 antagonist SCH23390 (0.01 µg stressin I + 120 ng SCH23390 per hemisphere, Figure 1I, mean ± SEM values are given in Supplementary Table S3, statistics in Supplementary Table S4). The D1 antagonist SCH23390 blocked both the behavioral effect of CRHR1 stimulation in the amygdala as well as the CRHR1-mediated increase in D1 binding. To assess whether amygdala D1 binding levels were correlated with anxiety-like behavior, we conducted correlation analyses across all amygdala nuclei and ITCs, and the main EPM behavioral readouts (percent time spent in the open arms and percent open-arm entries), pooling data across treatments (Supplementary Figure S1) and performing a False Discovery Rate (FDR) correction. D1 levels across most amygdala subregions were strongly inter-correlated (r ≈ 0.6–0.9), and the two EPM measures were highly correlated (r = 0.76). Both behavioral readouts resulted generally in negative correlations with D1 expression across nuclei (r ≈ −0.3 to −0.65). Notably, D1 levels in the medial paracapsular intercalated cells (Imp) exhibited the most robust relationship with anxiety-like behavior, representing the only region with a significant negative correlation with both EPM measures. D1 expression in the Ivp also showed significant negative correlations with percent time in the open arms. Correlations between D1 levels in both the BMA and MeA, but not the CeA (Benjamini–Hochberg adjusted p = 0.051), were also significantly negatively correlated with percent time in the open arms. For correlation matrix, see Supplementary Figure S1.

### Increase of stressin I-induced D1 binding is dependent on co-localized CRHR1 receptors

Male D1^Cre^-Crhr1^−/−^ (n = 14) and Crhr1^f/f^ (n = 11) mice were equipped with i. c.v. cannulae. One hour after i. c.v. injection of stressin I (0.5 µg in 2 µL), D1 binding sites were assessed by receptor autoradiography. Similar to the rat experiment, stressin I increased D1 binding sites only in amygdala regions of control Crhr1^f/f^ mice, while this effect was completely absent in D1^Cre^-Crhr1^−/−^ mice (mean ± SEM values and statistics are summarized in Supplementary Tables S5 and S6, respectively).

### Behavioral characterization of D1^Cre^-Crhr1^−/−^ mice

Conclusive proof of the suggested CRHR1-D1 receptor interaction cannot be obtained by pharmacological tools. We therefore employed D1^Cre^-Crhr1^−/−^ knockout mice. The generation of this transgenic mouse line has been descibed in Bernardi et al. (2017). A series of behavioral tests was performed to characterize the behavior of D1^Cre^-Crhr1^−/−^ mice and their Crhr1^f/f^ littermates under basal conditions (homecage locomotion, Open Field, EPM, and Dark-Light Box) and during stressed conditions (auditory fear conditioning).

**FIGURE 2:**
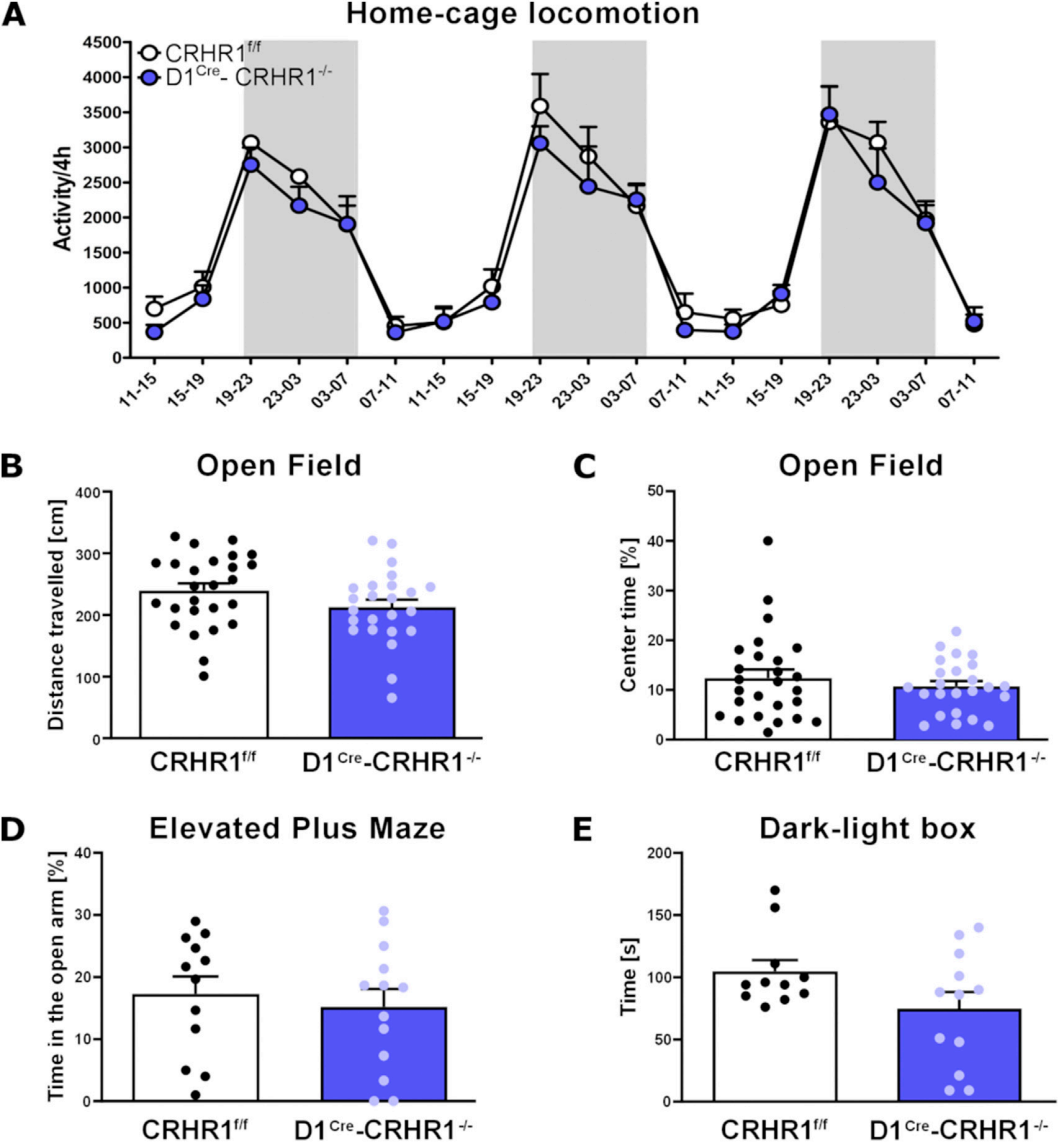
Measures of homecage and Open Field locomotion, EPM, and the Dark-Light test in the D1^Cre^-Crhr1^−/−^ mouse line. A set of behavioral tests were performed to address the basal behaviors in control Crhr1^f/f^ mice (white bars) and their specific knock-out D1^Cre^-Crhr1^−/−^ littermates (violet bars). **(A)** Homecage locomotion over 3 days for Crhr1^f/f^ mice (n = 5) and their D1^Cre^-Crhr1^−/−^ littermates (n = 4). Dark phases are shown with a grey background. Data are expressed as activity/4 h. **(B)** Total distance travelled (cm) during 15 min of Open Field in Crhr1^f/f^ mice (n = 26) and their D1^Cre^-Crhr1^−/−^ littermates (n = 24). **(C)** Time spent in the center of the arena (s) during 15 min of Open Field in Crhr1^f/f^ mice (n = 26) and their D1^Cre^-Crhr1^−/−^ littermates (n = 24). **(D)** Percentage of time spent in the open arms during 5 min of Elevated Plus Maze in Crhr1^f/f^ mice (n = 12) and their D1^Cre^-Crhr1^−/−^ littermates (n = 13). **(E)** Time spent in the light compartment during the 5 min in the Dark-Light box test (s) in Crhr1^f/f^ mice (n = 11) and their D1^Cre^-Crhr1^−/−^ littermates (n = 12). Data are shown as individual data points with the mean ± SEM.

One group of animals was measured for homecage locomotion over 3 consecutive days. Homecage locomotion did not differ between the two genotypes (D1^Cre^-Crhr1^−/−^(n = 4), CRHR1^f/f^ (n = 5), F [1,7] = 0.8, p > 0.05). As expected, mice displayed higher activity during the dark phase (repeated measures analysis showed a main effect for hours (F [17.119] = 39.9, p < 0.001, η^2^_p_ = 0.851) and lower activity during the light phase (Figure 2A).

Another group of animals (D1^Cre^-Crhr1^−/−^(n = 24), Crhr1^f/f^ (n = 26) were exposed to the Open Field. The total distance (cm) travelled during the 15 min trial did not differ between the genotypes (one-way ANOVA: F [1.48] = 2.5, p > 0.05, Figure 2B). Importantly, the percentage of time spent in the center of the arena, considered an index for anxiety, also did not differ between the genotypes (one-way ANOVA: F [1.49] = 0.6, p > 0.05, Figure 2C). Following Open Field testing, this group of animals was divided into two subgroups for the EPM and Dark-light tests (D1^Cre^-Crhr1^−/−^, n = 13; Crhr1^f/f^, n = 12) and fear conditioning (D1^Cre^-Crhr1^−/−^, n = 11; Crhr1^f/f^, n = 14).

In the EPM test, D1^Cre^-Crhr1^−/−^ (n = 13) and Crhr1^f/f^ (n = 12) mice did not differ in the time spent in the open arms during the 5-min session (one way ANOVA: main effect of genotype: F [1.23] = 0.3, p > 0.05, Figure 2D). In the Dark-Light box test, D1^Cre^-Crhr1^−/−^ and Crhr1^f/f^ (n = 12 and n = 11, respectively; two mice, 1 from each group, were removed from analysis because 1 escaped the apparatus during the test and 1 was found to be a significant outlier as revealed by Grubbs’ test) mice did not differ in the time spent in the light compartment during the 5-min session (one way ANOVA: F [1.21] = 3.3, p > 0.05, Figure 2E). These findings suggest that under basal conditions, locomotion and typical measures of anxiety are not affected by the knockdown of CRHR1 in D1-containing neurons.

**FIGURE 3:**
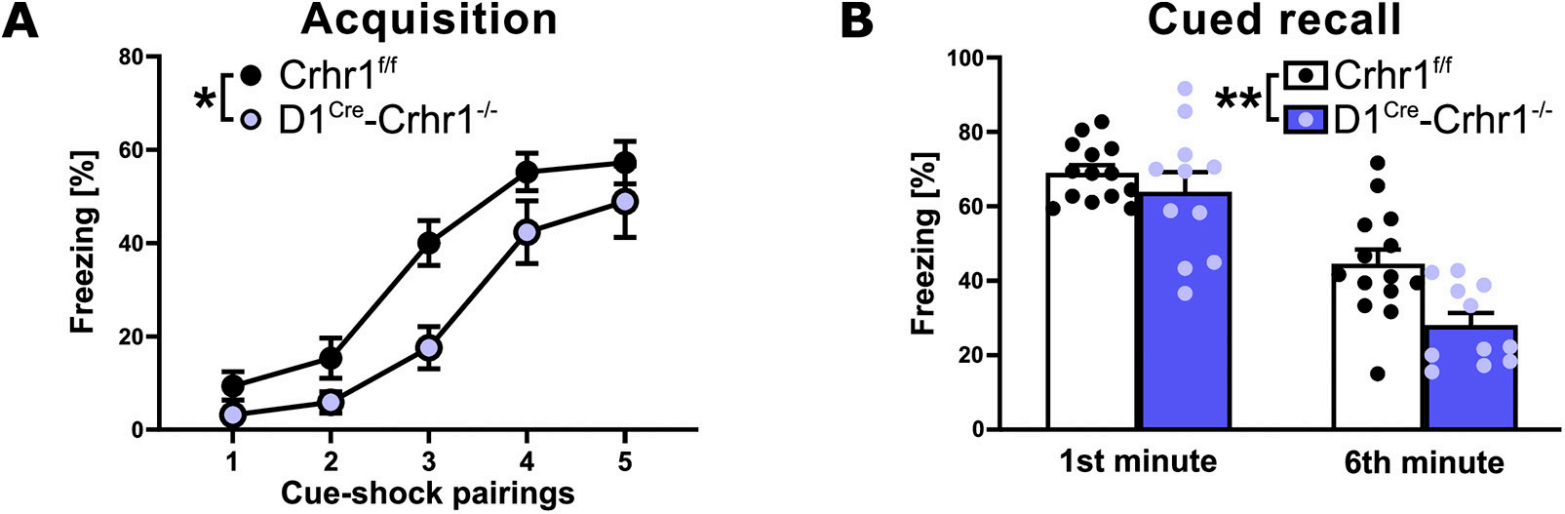
Auditory fear conditioning in the D1^Cre^-Crhr1^−/−^ mouse line. Auditory fear conditioning during acquisition **(A)** and cued recall **(B)** in Crhr1^f/f^ mice (white bars; n = 14)) and their D1^Cre^-Crhr1^−/−^ littermates (violet bars; n = 11). For acquisition, data are expressed as percentage of freezing time following each of the five cue-shock pairings of the 6-min session. For the recall test, data are expressed as percentage of freezing time during the first and sixth minute of the 6 min test. Data are shown as individual data points with the mean ± SEM. *p < 0.05, **p < 0.01, ***p < 0.001.

In the subset of animals tested for fear learning, D1^Cre^-Crhr1^−/−^ (n = 11) and Crhr1^f/f^ (n = 14) were tested in an auditory fear conditioning protocol, commonly used to investigate the responses to threatening and stressful stimuli. During the acquisition phase, although freezing increased with subsequent cue-shock pairings across the 6-min session for both groups, as is typical in fear conditioning, D1^Cre^-Crhr1^−/−^ demonstrated a lower percentage freezing relative to Crhr1^f/f^ mice (Figure 3A; repeated measures ANOVA, main effect of pairing: F (2.6, 60.2) = 59.1, p < 0.001, η^2^_p_ = 0.720); main effect of genotype: F (1.23) = 7.7, p = 0.011, η^2^_p_ = 0.252); pairing × group interaction: F (2.6, 60.2) = 1.3, p > 0.05). A repeated measures ANOVA revealed no difference between genotypes during contextual recall, as measured by an analysis of the percentage freezing during the first and sixth minute of the 6-min recall test (main effect of minute: F (1.23) = 0.03, p > 0.05; main effect of genotype: F (1.23) = 0.004, p > 0.05; minute × group interaction: F (1.23) = 0.004, p > 0.05; *data not shown*). In contrast, a repeated measures ANOVA revealed that D1^Cre^-Crhr1^−/−^ demonstrated a lower percentage freezing during amygdala-dependent cued recall relative to Crhr1^f/f^ mice (Figure 3B), as measured by an analysis of the percentage freezing during the first and sixth minute of the 6-min recall test (main effect of minute: F (1.23) = 82.7, p < 0.001, η^2^_p_ = 0.782; main effect of genotype: F (1.23) = 7.1, p = 0.014, η^2^_p_ = 0.252; minute × group interaction: F (1.23) = 2.9, p > 0.05). These findings suggest impaired auditory fear learning and a subsequently lower fear recall.

### Dysregulated D1-CRHR1 interaction in alcohol dependence

**FIGURE 4:**
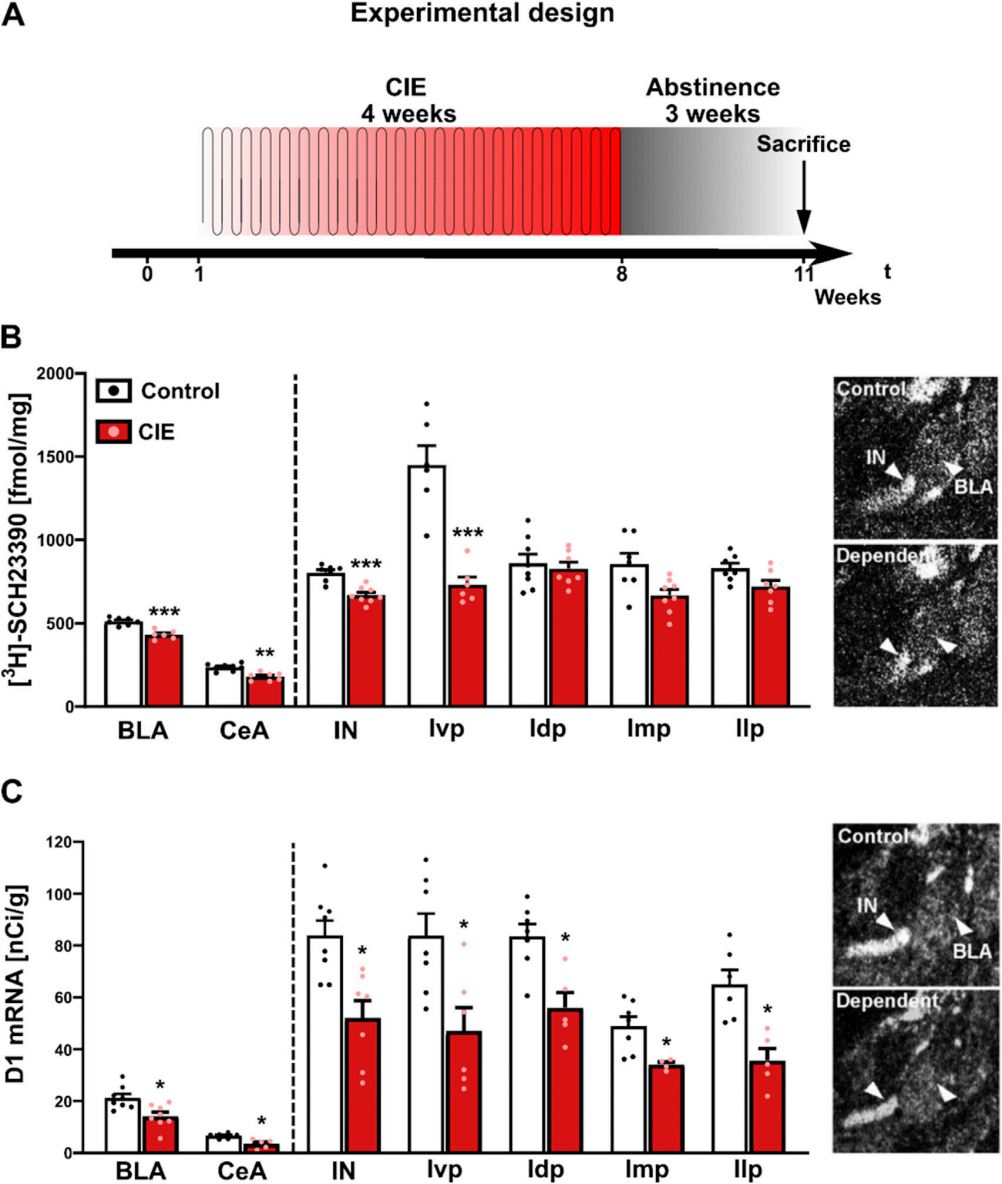
D1 protein and Drd1 mRNA expression levels were strongly decreased in the amygdala regions of abstinent rats. Dependence was induced by cyclic intermittent alcohol vapor exposure (CIE) in rats. Three-week abstinent rats were assessed for D1 expression. **(A)** Experimental time line. **(B)** D1 receptor binding sites (expressed in fmol/mg) were analyzed by 10 nM [3H]-SCH23390 for saturated receptor autoradiography. Non-specific binding was measured by adding 1 µM SKF (not shown). **(C)** Expression patterns are similar for Drd1 mRNA *in situ* hybridization. Non-specific hybridization was determined by a sense riboprobe (not shown). The small panels on the right show the D1 distribution in the analyzed BLA, CeA and ITC clusters for receptor autoradiography **(B)** and Drd1 *in situ* hybridization **(C)** assays in controls and dependent CIE rats. Arrowheads indicate localization of IN and BLA. Data are expressed as means ± SEM with individual data points (receptor autoradiography: n = 4–8/group; Drd1 *in situ* hybridization: n = 6–8/group). *p < 0.05, **p < 0.01, ***p < 0.001. BLA, basolateral amygdala; CeA, central amygdala; Idp, dorsal paracapsular intercalated cells; Ilp, lateral paracapsular intercalated cells; Imp, medial paracapsular intercalated cells; IN, intercalated amygdaloid nucleus, main part; Ivp, ventral paracapsular island.

Dependence was induced in a cohort of rats (n = 8/group) using the CIE procedure (Figure 4A). During exposure, blood alcohol concentrations (BAC) were 250–300 mg/dL. After 3 weeks of abstinence, rats were used for D1 autoradiography and *Drd1 in situ* hybridization.

Control rats showed a similar D1 distribution as in the previous experiments. However, in CIE rats, we found a pronounced reduction of D1 receptors across amygdala regions, with the highest effect in the Ivp, both on the receptor binding (Figure 4B) and transcription levels (Figure 4C). Data and statistics are summarized in Supplementary Table S7 (for D1 receptor binding) and in Supplementary Table S8 (for *Drd1* mRNA). We also checked whether this reduction was due to structural changes within the amygdala region. Spine density and spine length within the amygdala of CIE to alcohol and air-exposed control rats were measured according von Bohlen und Halbach et al. (2006). No significant changes were observed in the CEA, BLA and MeA (*data not shown*; data are given in Supplementary Table S9).

**FIGURE 5:**
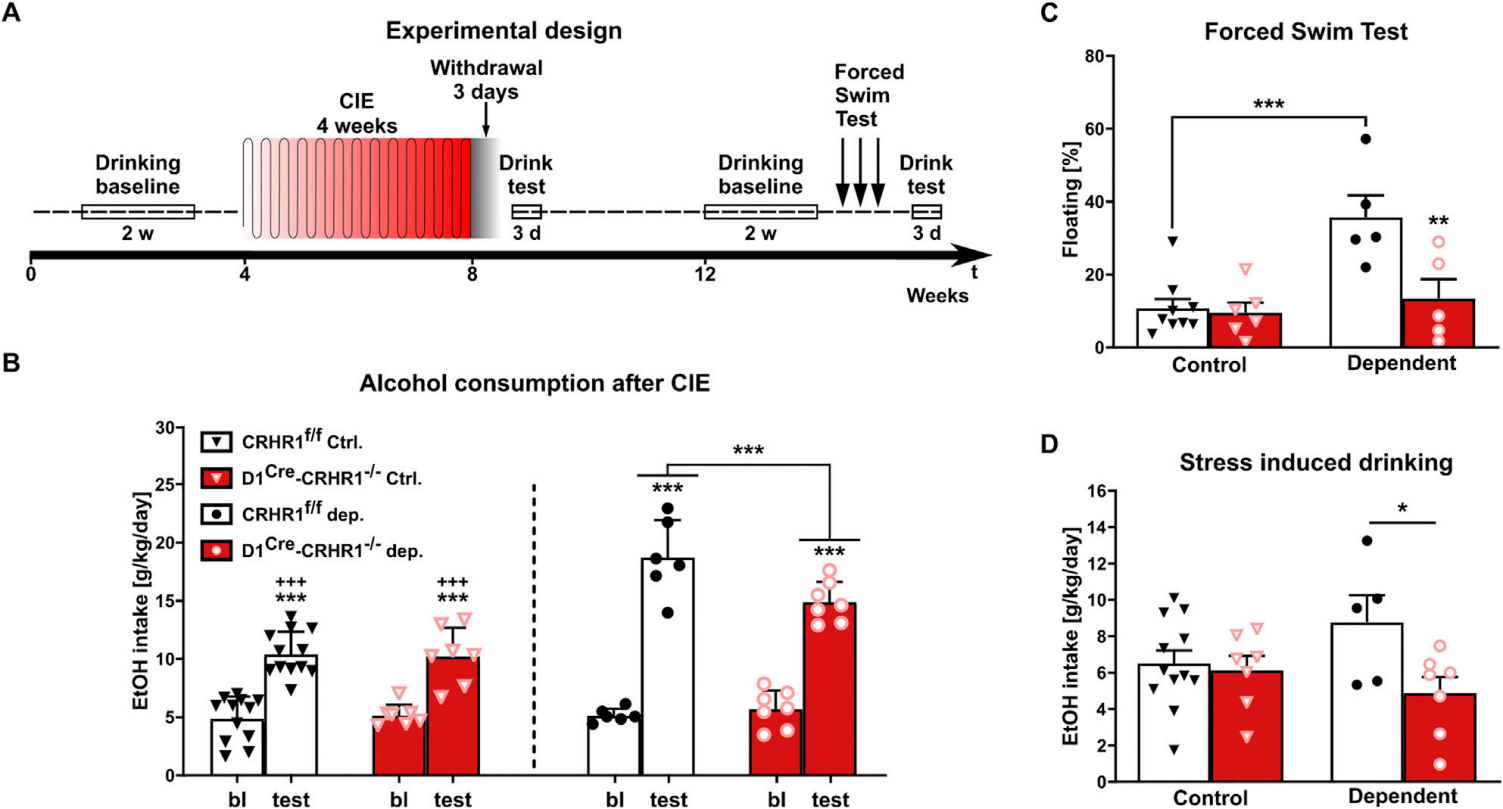
Dependent D1^Cre^-Crhr1^−/−^ mice (n = 5–7) displayed a lower alcohol intake and vulnerability to stress compared to their dependent Crhr1^f/f^ littermates (n = 5–12). **(A)** Experimental time line. **(B)** Voluntary alcohol consumption (g/kg/day) in Crhr1^f/f^ and D1^Cre^-Crhr1^−/−^ mice 3 days after ethanol exposure (CIE) and air controls compared to baseline (bl) drinking prior to exposure. Alcohol intake is expressed in g/kg/day. **(C)** Percentage of floating during the first day of the Forced Swim Stress in control and dependent animals. **(D)** Alcohol consumption during the first day after repeated FSS. All data are shown as means ± SEM with individual data points. *p < 0.05, **p < 0.01, ***p < 0.001.

Next, the relevance of CRHR1-D1 interactions for alcohol behaviors was investigated in D1^Cre^-Crhr1^−/−^ mice (timeline shown in Figure 5A). Comparing baseline and mean drinking levels during three consecutive days of two-bottle free-choice paradigm following CIE, we found a significant genotype x CIE × condition interaction (F [1.28] = 4.3, p = 0.047, η^2^_p_ = 0.191). Newman-Klaus *post hoc* analysis indicated that baseline alcohol consumption did not differ among the four groups (Figure 5B). Furthermore, after 4 weeks of daily CIE followed by 3 days of withdrawal, alcohol-dependent mice increased their daily alcohol intake (p < 0.001) as expected from the previous studies. However, D1^Cre^-Crhr1^−/−^ mice consumed significantly less alcohol than controls (p < 0.001, Figure 5B; Supplementary Table S10). Notably, air-exposed mice increased their alcohol consumption, which reflects the well-documented alcohol deprivation effect, in which renewed access to alcohol after a period of deprivation leads to a pronounced temporary increase in voluntary alcohol intake (Spanagel, 2017), but this effect was independent of genotype (Figure 5B; Supplementary Table S10). Potential confounds for the increased alcohol intake, such as differences in alcohol metabolism or severity of alcohol withdrawal between the genotypes, were excluded.

Alcohol metabolism was evaluated at the same time points as for the withdrawal signs: BAC immediately after the intoxication correspond to 188.15 ± 2.45 mg/dL in Crhr1^f/f^ mice, while in D1^Cre^-Crhr1^−/−^ mice was 209.45 ± 9.85 mg/dL. During the following 4 and 8 h, BAC decreased until reaching values of 3.90 ± 0.20 and 7.95 ± 0.55 mg/dL 12 h after the last exposure, showing no differences between the genotypes (F [1,2] = 0.2, p > 0.05).

After 1 month of homecage drinking, all animals reached a stable comparable alcohol consumption that was independent of genotype and history of dependence (two-way ANOVA genotype:F [1.29] = 0.01, p > 0.05; treatment: F [1.29] = 0.4, p > 0.05; genotype x treatment:F [1.29] = 0.02, p > 0.05). Stress coping is typically altered in AUD, resulting in a hypersensitivity to stressful stimuli. We tested the stress coping style of the mice using exposure to repeated swim stress for 3 consecutive days. Interestingly, only CIE-treated Crhr1^f/f^ mice showed a behavioral despair response, measured as increased floating time and increased post-stress alcohol consumption (Figure 5C; 2-way ANOVA floating: genotype F [1.21] = 8.5, p = 0.008, η^2^_p_ = 0.288, CIE F [1.21] = 12.8, p = 0.002, η^2^_p_ = 0.379, interaction F [1.21] = 6.8, p = 0.016, η^2^_p_ = 0.246). Based *post hoc* tests following a genotype × CIE interaction trend (2-way ANOVA alcohol intake: F [1.27] = 3.4, p = 0.07, η^2^_p_ = 0.113), the drinking behavior of CIE D1^Cre^-Crhr1^−/−^ mice was indistinguishable from mice without a history of alcohol dependence (p > 0.05), and reduced relative to dependent Crhr1^f/f^ mice (p = 0.034) (Figure 5D).

### D1 and CRHR1 are co-localized within the amygdala

**FIGURE 6:**
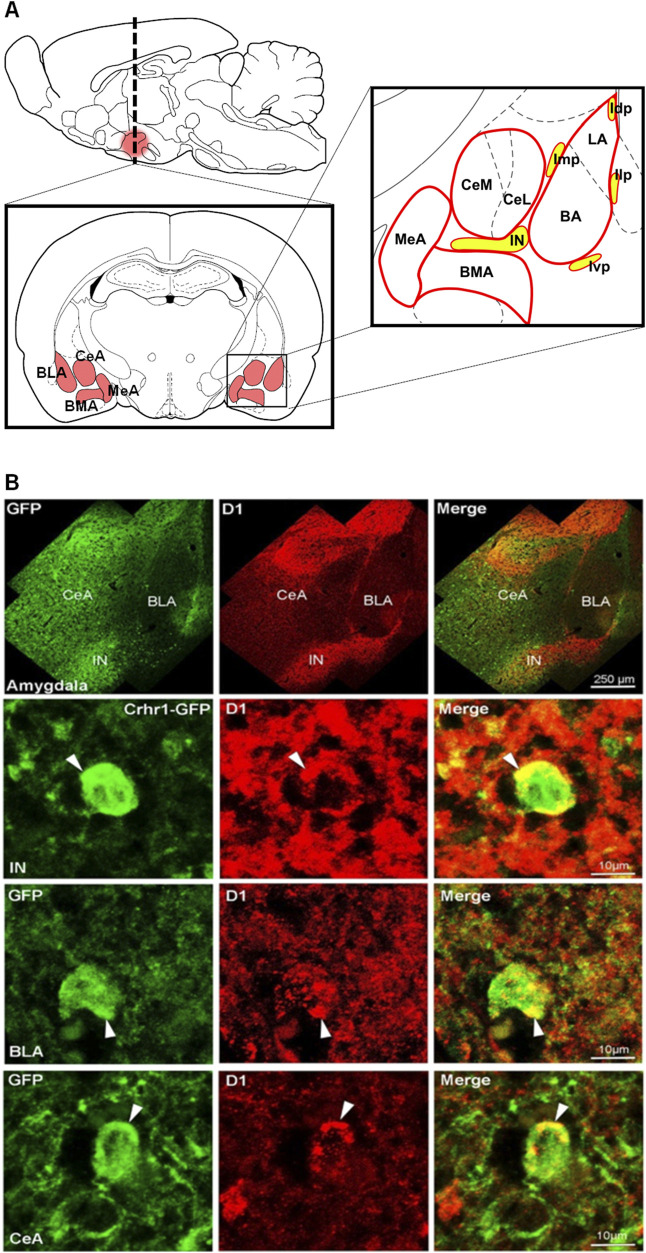
D1-CRHR1 co-localization in the amygdala brain region. **(A)** Schematic representation of the different amygdala nuclei and intercalated paracapsular cell clusters. **(B)** (Upper panel) Confocal images present an overview of the entire amygdala obtained by double-immunostaining for GFPir (green), D1ir (red) and co-localized receptors (yellow) in merged channels. (Lower panels) Amygdala sections co-stained for GFP and D1 reveal substantial overlap in the IN, intercalated amygdaloid nucleus, main part, and to a lesser extent in basolateral amygdala (BLA) and central amygdala (CeA). White arrow points to co-localized receptors. Ivp, ventral paracapsular island; Imp, medial paracapsular intercalated cells; Idp, dorsal paracapsular intercalated cells; Ilp, lateral paracapsular intercalated cells; BMA, basomedial amygdala; MeA, medial amygdala.

We performed double fluorescence immunohistochemistry for D1 and CRHR1 using *Crhr1*-GFP driver mice. Fluorescence immunohistochemistry on coronal amygdala brain sections were performed according to (Bernardi et al., 2017; Broccoli et al., 2018). CRHR1 (GFP-ir, green color) is visualized in the nucleus and cytoplasm of the neurons, while D1-ir is shown in red color (Figure 6). In line with previous studies, D1-ir is expressed thoughout the amygdala, with the highest expression found in ITCs, while CRHR1-GFP is evenly expressed in most of the amygdala regions including ITCs. Co-localization of GFP-ir and D1-ir is visualized in orange-to yellow color-labelled merged images. Extensive co-localization was found in the main part intercalated amygdaloid nucleus (IN), lateral paracapsular intercalated cells (Ilp), medial paracapsular intercalated cells (Imp), and to a lesser extent other amygdala subregions (e.g., BLA).

### CRHR1 enhances DA-induced hyperpolarization in the ITCs

Building on the work of Marowsky et al. (2005) showing that DA hyperpolarizes ITCs *via* D1 receptors, we investigated the effects of stressin I on DA by patch-clamp recordings. Acutely-prepared amygdala slices from *GAD67*-GFP mice were used, in which ITC cells can be readily identified based on the densely packed GABAergic neurons (Millhouse, 1986; Pare and Smith, 1993).

**FIGURE 7:**
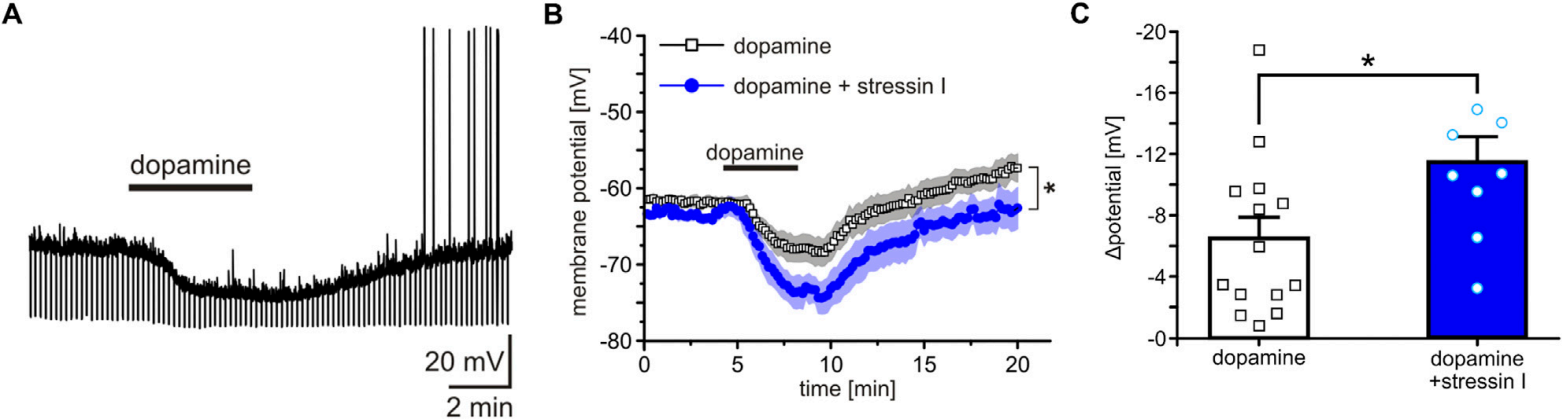
Co-activation of CRHR1 and D1 enhances hyperpolarization of amygdala ITCs. **(A)** Representative current-clamp recording from a single D1-containing ITC. Bath application of 20 µM DA induced a hyperpolarization of the membrane potential. **(B)** Quantification of DA-induced changes in membrane potential in ITCs treated either with DA alone (white) or with DA after pre-incubation with 250 nM stressin I (blue). **(C)** Quantification of the maximal effect shows a significant increase of the DA-induced hyperpolarization following stressin I pre-incubation. *p < 0.05, **p < 0.01, ***p < 0.001.

Bath application of 20 µM DA induced a hyperpolarization of the ITCs recorded in current clamp at a membrane potential between −60 and −65 mV (Figure 7A). Interestingly, after pre-incubation of slices with a highly selective CRHR1 agonist, stressin I (250 nM), DA-induced changes of the membrane potential were significantly increased (RM-ANOVA time × substance interaction: F (119, 2,499) = 1.276; p = 0.026; partial η^2^ = 0.092, DA, n = 14, DA + stressin I, n = 8; Figure 7B). The maximal change of the membrane potential (Δ potential) was −6.5 ± 1.38 mV by DA alone and 11.51 ± 1.65 mV after stressin I treatment (U-test: U = 26; z-score: 2.299; p = 0.021; effect size r = 0.56, Figure 7C).

### D1 and CRHR1 form heteroreceptor complexes *in vitro*

**FIGURE 8:**
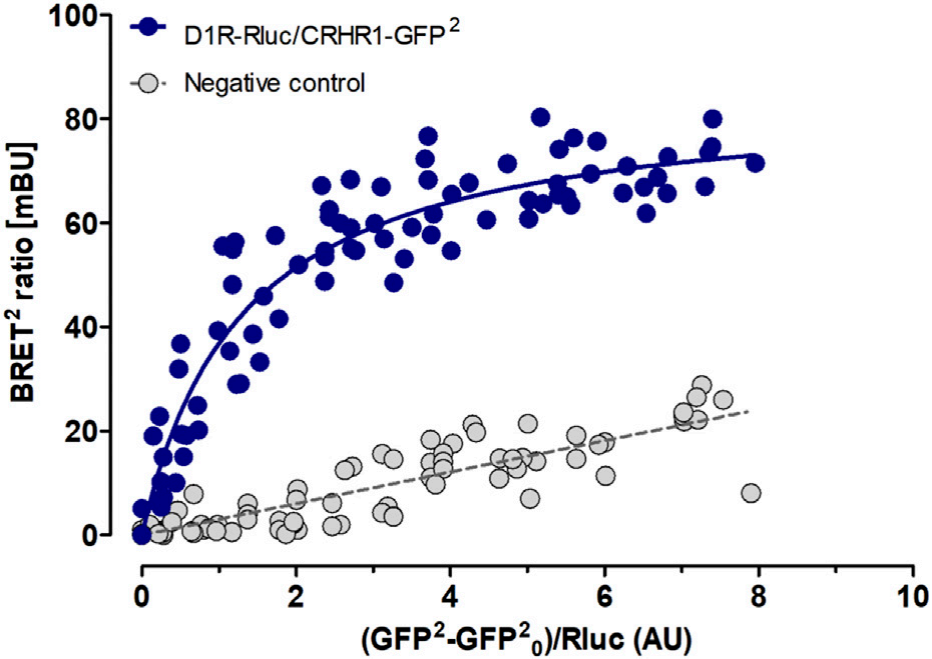
D1 and CRHR1 form heteroreceptor complexs *in vitro*. BRET assay shows specific DA D1 and CRHR1 interaction in HEK293T cells co-transfected with a constant amount of D1-Rluc plasmid and increasing amount of the CRHR1-GFP2 plasmid (blue dots). Cells individually expressing D2L-Rluc or NTS1R-GFP2 were used as a negative control (grey dots). The fluorescence value obtained from the GFP2, plotted on the X-axis, is normalized with the luminescence value of Rluc expression 10 min after coelenterazine incubation. Data are expressed in mean ± SEM (n = 4, in 8 replicates). The D1-CRHR1 curve fitted better to a saturation curve than to a linear regression as found with mixed pools of cells individually expressing D1-Rluc + CRHR1-GFP2 (F test, p < 0.001). Data are expressed as mean ± SEM; n = 4.

The combined evidence of our study suggests that the dependent phenotype depends on D1-CRHR1 interactions in the amygdala. Here we used BRET to demonstrate the possibility of direct physical interactions between D1 and CRHR1, i.e., of D1-CRHR1 heteroreceptor complexes. HEK293T27 cells were co-transfected with a constant amount of D1-Rluc plasmid and increasing amount of the CRHR1-GFP^2^ plasmid. As a negative control with no possibility for heteromer formation, cells expressing either D1-Rluc or CRHR1-GFP^2^ plasmid were mixed together. A D1-CRHR1 curve fitted better to a saturation curve than to a linear regression as found in the negative control (F-test: p < 0 .001), which revealed a high affinity between the two receptors. Furthermore, high BRET_max_ (mean ± SEM: 84.98 ± 2.95) and low BRET_50_ (mean ± SEM: 1.34 ± 0.16) values together suggested that the saturation level was reached with a fast rate, which confirms the elevated affinity between D1 and CRHR1 (Figure 8).

## Discussion

In this study we provide evidence across multiple animal cohorts of D1-CRHR1 interactions in the amygdala that mediate anxiogenic responses. Importantly, these interactions are specifically found in the amygdala but not in other brain regions such as BNST and striatum that are known to co-express these receptors. In alcohol dependence, this interaction seems to be critically involved in maladaptive stress coping, excessive alcohol consumption, and stress-induced alcohol intake.

To demonstrate that D1-CRHR1 interactions exist *in vivo*, we used classical pharmacological and advanced transgenic approaches. Stimulation of CRHR1 *via* i. c.v. injection of the highly selective agonist stressin I strongly upregulated D1 binding sites in all amygdala regions, but especially within the ITCs. The effect obtained by CRH treatment was not as strong as that of stressin I. This might be due to different affinities of the ligands. CRH, a natural neuropeptide, binds to both CRHR1 (Ki = 0.95 nM) and CRHR2 (Ki = 13 nM) (Donaldson et al., 1996), while stressin I shows high specificity for CRHR1 (Ki = 1.5 nM) over CRHR2 (Ki = 224 nM) (Rivier et al., 2007; Rivier et al., 2002). Thus, local CRHR2 may have affected the availability of CRH receptor competition or acted on the signaling level to reduce CRHR1 actions (Grammatopoulos, 2012), consistent with its role in attenuating responses to stress (Kishimoto et al., 2000; Bale et al., 2000; Valdez et al., 2003). CRHR1-mediated D1 binding was not found outside the amygdala, although co-localization of D1 and CRHR1 binding sites exists in other extra-amygdala brain regions, such as the BNST and VTA (Bernardi et al., 2017). Anatomical specificity was demonstrated by intra-amygdala injection of stressin I. Site-specific injections of stressin I induced an increase in D1 binding sites in the amygdala region similar to those demonstrated with systemic administration. The stressin I-induced increase in D1 was fully blocked by co-treatment with the D1 antagonist SCH23390, thus confirming a strong impact of CRHR1 on D1 availability.

In parallel, intra-amygdala stressin I administration resulted in an expected increase in anxiety in the EPM that was blocked by co-treatment with SCH23390. The activation of CRHR1 by stressin I resulting in anxiogenic behavior has been consistently reported (Smagin et al., 2001; Bruchas et al., 2009). Furthermore, D1 blockade by injection of SCH23390 into the amygdala/ITC has been shown to result in an anxiolytic effect in the EPM (de la Mora et al., 2005), consistent with our findings. D1 effects on anxiety-related behavior have been further shown in studies using different behavioral paradigms, including fear-potentiated acoustic startle response (Lamont and Kokkinidis, 1998), fear conditioning (Guarraci et al., 1999; El-Ghundi et al., 2001), and the Dark-Light box (de la Mora et al., 2005). Interestingly, we further found in a correlation analysis that D1 binding in several regions in the amygdala, as well as Imp and Ivp, were significantly negatively correlated with percent time in the open arms in the EPM, suggesting D1 is proportional to stress intensity. DA signaling has previously been shown to positively correlate with stimulus salience and, as already discussed, likely acts through D1 receptors in the amygdala/ITCs to mediate anxiety-like behavior (Inglis and Moghaddam, 1999; de la Mora et al., 2005; Wang and Tsien, 2011; Asede et al., 2022). Although the DA system is also involved in the regulation of motor behavior, the site-specific blockage of D1 in the amygdala did not alter locomotion activity. Indeed, the number of total entries into the closed arm of the EPM, a measure of locomotor activity, did not differ between the treatment groups. Thus, our results demonstrate that stimulation of CRHR1 mediates the responses to stressful stimuli at least in part by synergistic activation of D1 in the amygdala. Together, these findings suggest that CRHR1 mediates anxiogenic-like responses at least in part *via* D1.

In further evidence of a D1-CRHR1 interaction, i. c.v. stressin I increased D1 binding sites in amygdala regions in control Crhr1^f/f^ mice, similar to our findings in rats, but had no effect on D1 binding sites in D1^Cre^-Crhr1^−/−^ knockout mice, thus strengthening our findings by providing cross-species confirmation of these interactions. Behaviorally, under basal, non-stressed conditions, D1^Cre^-Crhr1^−/−^ and Crhr1^f/f^ mice did not differ in homecage locomotion or Open Field activity, and showed no significant differences in anxiety-related responses in the EPM or Dark-Light box. In contrast, under the stressed conditions of auditory fear conditioning, in which fear learning and responses are highly dependent on amygdala circuitry (Herry and Johansen, 2014; Trent et al., 2025), D1^Cre^-Crhr1^−/−^ mice showed an impaired acquisition of auditory fear conditioning that was also reflected in an impairment in subsequent auditory cued recall. Consistent with these findings, previous studies have demonstrated that aversive stimuli such as foot shocks induce activation of CRH activity in the amygdala that facilitates fear learning *via* CRHR1, the disruption of which results in impaired fear acquisition and/or expression (Merali et al., 1998; Bakshi et al., 2002; Isogawa et al., 2013; Sanford et al., 2017). In addition, increased DA release in the amygdala, specifically acting *via* D1 receptors, is critical for normal fear acquisition by integrating aversive stimuli such as footshocks and neutral stimuli associated with these events (Lee et al., 2017; Zafiri and Duvarci, 2022; Hamati et al., 2024). As demonstrated in the current manuscript and elsewhere, D1 receptors are highly expressed in ITCs (Asede et al., 2021; 2022; Fuxe et al., 2003), and thus D1 receptors co-localized with CRHR1 in ITCs may contribute to the modulation of fear learning demonstrated here. Nonetheless, our findings are indicative of impaired amygdala-specific emotional response learning that suggests the presence of an interaction between CRHR1 and D1 in the amygdala region that requires stimulation of the CRH system.

We also tested D1-CRHR1 interactions in alcohol-dependent rats. In protracted abstinence, D1 expression was decreased within the amygdala and ITCs. These data thus indicate a neuroadaptative loss of D1 receptor signaling in ITC cell populations that likely co-express D1 and CRHR1. A reduction of D1 has been found in brain tissue of deceased AUD patients, emphasizing the translational value of these findings (Hirth et al., 2016). We also found that the CIE procedure had no effect on the number of dendritic spines and dendritic spine density within the CeA, MeA and BLA, suggesting that the reduction in D1 receptors is unlikely due to structural changes. In the same animal model, we previously found an increase in tonic DA levels in the nucleus accumbens, as demonstrated by *in vivo* microdialysis (Hirth et al., 2016; Hansson et al., 2019), a finding confirmed in extracellular fluid *via ex vivo* mass spectrometry (Meinhardt et al., 2015). These data imply an increased DA drive in the amygdala in the dependent state (i.e., hyper-dopaminergic state), which leads to a counteradaptive decrease in D1 binding sites (Hansson et al., 2019).

To investigate the impact of a D1-CRHR1 interaction on the dependence-induced phenotype, alcohol consumption in D1^Cre^-Crhr1^−/−^ mice and their littermate controls (Crhr1^f/f^) was investigated using a two-bottle free-choice paradigm. After 3 days of withdrawal, all exposed mice increased their daily alcohol intake, consistent with previous studies in mice (Gilpin et al., 2009; Lopez and Becker, 2005). However, mice lacking CRHR1 in D1-containing neurons consumed significantly less alcohol than control mice. To further identify the involvement of a D1-CRHR1 interaction in the regulation of stress during alcohol dependence, the CRH system was again challenged by repeated FSS, and alcohol intake was monitored during the following days. Dependent D1^Cre^-Crhr1^−/−^ mice showed a reduced percentage of floating-time during the test and subsequently less stress-induced alcohol intake, clearly indicating a diminished vulnerability to stress compared to controls. As demonstrated in previous studies, CRH-driven hypersensitivity is crucial in the development of alcohol dependence and withdrawal, causing relapse and negative affective states (Lovinger and Roberto, 2023; Heilig and Koob, 2007; Sommer et al., 2008). Our study further confirms this concept, as alcohol intake did not differ between the groups during baseline conditions, and following repeated stimulation of CRHR1 by FSS, both non-dependent and dependent D1^Cre^-Crhr1^−/−^ mice showed no increase in alcohol consumption. Thus, dependent D1^Cre^-Crhr1^−/−^ mice are less vulnerable to stress and less responsive to aversive stimuli during alcohol dependence.

Our experiments with D1^Cre^-Crhr1^−/−^ mice suggest that the interaction of D1 and CRHR1 receptors is likely dependent on co-localized receptors. We verified this with double fluorescence immunohistochemistry for D1 and CRHR1 using *Crhr1*-GFP driver mice. D1 is highly and abundently expressed in the ITCs, where a high degree of co-localized receptors has been observed. Thus, most of the pharmacological effects described above appear to be mediated by co-localized receptors in the ITC.

Further functional evidence of DA-CRHR1 interactions has been provided by *ex-vivo* recordings using a GAD67 fluorescence reporter strategy for patch-clamp analysis in acutely prepared amygdala slices. DA induced a hyperpolarization of the ITCs, which is most likely mediated by D1 receptors (Marowsky et al., 2005). Remarkably, co-treatment of DA with stressin I further enhanced and prolonged the hyperpolarization of these cells. Thus, under stressful conditions, the activation of both D1 and CRHR1 disinhibits the BLA and the CeA, leading to an enhanced response to a stressful stimulus.

Our initial BRET assay confirms a high affinity between D1 and CRHR1, demonstrating the fast formation of a D1-CRHR1 heterocomplex *in vitro*, at a similar rate as demonstrated for adenosine A2A and DA D2 receptor heteromers (Hillion et al., 2002). The functional relevance of GPCR homo- or heteromerization has been previously assessed, demonstrating its impact on receptor trafficking (Terrillon and Bouvier, 2004), ligand affinity (El-Asmar et al., 2005) and receptor signaling (Lohse, 2010). The capability of D1 and CRHR1 receptors to heteromerize has not yet been demonstrated *in vivo*.

In conclusion, our results support the well-established role of amygdala CRHR1 systems in mediating anxiety- and stress-related responses and their overactivation in alcohol dependence. Our findings unveil a novel mechanism involving D1–CRHR1 interactions within amygdala-ITC pathways. We show that during protracted abstinence, diminished D1 binding in the amygdala likely compensates for elevated CRHR1 levels and heightened dopaminergic activity, together facilitating stress-induced alcohol drinking and perpetuating the chronic negative affective state that drives relapse. These findings highlight D1–CRHR1 receptor crosstalk as a promising polypharmacological target to prevent anxiety-related relapse in AUD. Importantly, given the dynamic fluctuation of the dopaminergic system - and especially D1 receptor availability - during abstinence in both humans and rodent models (Hirth et al., 2016; Hansson et al., 2019), this mechanism may be modulated across both hypo- and hyper-dopaminergic states. Finally, while preclinical evidence consistently implicates CRHR1 in stress- and dependence-driven alcohol behaviors, clinical translation has so far been unsuccessful: CRHR1 antagonists such as pexacerfont and verucerfont have failed to attenuate craving or relapse in human trials (Kwako et al., 2015; Schwandt et al., 2016). Our findings suggest that targeting receptor co-localization and functional interactions, rather than CRHR1 in isolation, may provide a new translational strategy to overcome previous roadblocks in therapeutic development.

## Data Availability

The datasets presented in this study can be found in online repositories. The names of the repository/repositories and accession number(s) can be found in the article/Supplementary Material.
