# Supplementary material for: Reducing stress and alcohol-related behaviors by targeting D1-CRHR1 receptor interactions in the amygdala
Source: Front Pharmacol. 2025 Oct 16;16:1677510. doi: 10.3389/fphar.2025.1677510 (PMC12571840; doi:10.3389/fphar.2025.1677510)
Supplement: Supplementary file 1 [file Supplementaryfile1.docx]

**SUPPLEMENTARY INFORMATION**

**Table S1. Effects of CRH and stressin I on D1 receptor binding in different rat brain regions.** [^3^H]-SCH23390 binding 1 hour after i.c.v. injection of CRH (2µg/2µl injection volume), stressin I (4µg/2µl injection volume) and CSF (2µl injection volume) in rats. D1 binding data are shown as means±SEM. IN, intercalated amygdaloid nucleus, main part; Ivp, ventral paracapsular island; Imp, medial paracapsular intercalated cells; Idp, dorsal paracapsular intercalated cells; Ilp, lateral paracapsular intercalated cells; BLA, basolateral amygdala; CeA, central amygdala; BMA, basomedial amygdala; MeA, medial amygdala; CPu, caudate putamen; Cg, cingulate cortex; PrL, prelimbic cortex; IL, infralimbic region; M1, primary motor cortex; BNST, bed nucleus of the stria terminalis; SNR, substantia nigra reticulata.

| **Region** | | **CSF** | | **CRH** | | **stressin I** | |
| --- | --- | --- | --- | --- | --- | --- | --- |
|  |  | **Mean ± SEM**  **[fmol/mg]** | **N** | **Mean ± SEM**  **[fmol/mg]** | **N** | **Mean ± SEM**  **[fmol/mg]** | **N** |
| **Amygdala** | **IN** | 431.55 ± 32.02 | 6 | 654.24 ± 79.29 | 6 | 746.38 ± 29.92 | 4 |
|  | **Ivp** | 911.54 ± 18.07 | 5 | 1297.15 ± 38.03 | 6 | 1391.83 ± 97.10 | 6 |
|  | **Imp** | 731.29 ± 34.72 | 4 | 1199.18 ± 194.42 | 5 | 1012.28 ± 93.94 | 5 |
|  | **Idp** | 489.09 ± 27.93 | 5 | 699.76 ± 38.67 | 5 | 895.95 ± 61.30 | 5 |
|  | **Ilp** | 706.04 ± 34.70 | 5 | 818.71 ± 16.16 | 3 | 787.86 ± 59.05 | 3 |
|  | **BLA** | 339.35 ± 26.33 | 6 | 538.72 ± 25.21 | 5 | 611.55 ± 57.40 | 6 |
|  | **CeA** | 100.34 ± 8.52 | 6 | 159.62 ± 11.90 | 5 | 210.05 ± 9.55 | 6 |
|  | **BMA** | 171.10 ± 7.83 | 5 | 289.38 ± 4.21 | 5 | 321.48 ± 19.80 | 6 |
|  | **MeA** | 113.26 ± 11.25 | 6 | 185.98 ± 16.90 | 5 | 180.38 ± 9.55 | 6 |
| **Extra-amygdala regions** | **CPu** | 2045.81 ± 56.03 | 6 | 1753.41 ± 164.10 | 5 | 2092.08 ± 60.89 | 5 |
|  | **Cg** | 69.11 ± 10.35 | 4 | 71.03 ± 3.96 | 5 | 63.20 ± 11.75 | 4 |
|  | **PrL** | 94.83 ± 13.36 | 5 | 104.67 ± 8.17 | 5 | 93.54 ± 6.41 | 6 |
|  | **IL** | 162.40 ± 12.00 | 4 | 145.57 ± 19.73 | 4 | 135.28 ± 15.57 | 5 |
|  | **M1** | 27.29 ± 3.94 | 5 | 27.70 ± 3.44 | 5 | 24.28 ± 5.13 | 3 |
|  | **BNST** | 97.78 ± 5.73 | 5 | 94.72 ± 11.27 | 5 | 89.67 ± 5.75 | 5 |
|  | **SNR** | 1113.36 ± 148.20 | 5 | 874.39 ± 151.41 | 5 | 1153.58 ± 277.01 | 6 |

**Table S2. Regions with statistically significant effects on D1 receptor binding after i.c.v. injection of CRH and stressin I.** [^3^H]-SCH23390 binding 1 hour after i.c.v. injection of CRH (2µg/2µl injection volume), stressin I (4µg/2µl injection volume) and CSF (2µl injection volume) in rats. IN, intercalated amygdaloid nucleus, main part; Ivp, ventral paracapsular island; Imp, medial paracapsular intercalated cells; Idp, dorsal paracapsular intercalated cells; Ilp, lateral paracapsular intercalated cells; BLA, basolateral amygdala; CeA, central amygdala; BMA, basomedial amygdala; MeA, medial amygdala; CPu, caudate putamen; Cg, cingulate cortex; PrL, prelimbic cortex; IL, infralimbic region; M1, primary motor cortex; BNST, bed nucleus of the stria terminalis; SNR, substantia nigra reticulata.

| **Region** | | **F-value** | **p-value** | **CRH**  **vs CSF** | **stressin I**  **vs CSF** | **CRH**  **vs stressin I** |
| --- | --- | --- | --- | --- | --- | --- |
| **Amygdala** | **IN** | [2, 13]=7.71 | 0.006 | 0.012* | 0.003** | 0.303 |
|  | **Ivp** | [2, 14]=14.48 | 0.0004 | 0.001** | 0.0001*** | 0.305 |
|  | **Imp** | [2, 11]=2.83 | 0.102 | - | - | - |
|  | **Idp** | [2, 12]=20.59 | 0.0001 | 0.006** | 0.00003*** | 0.009** |
|  | **Ilp** | [2, 8]=2.34 | 0,159 | - | - | - |
|  | **BLA** | [2, 14]=12.50 | 0.0008 | 0.004** | 0.0003*** | 0.236 |
|  | **CeA** | [2, 14]=32.70 | 0.00001 | 0.00096*** | 0.00000*** | 0.003** |
|  | **BMA** | [2, 13]=19.96 | 0.000009 | 0.00005*** | 0.000003*** | 0.122 |
|  | **MeA** | [2, 14]=10.82 | 0.001 | 0.001** | 0.002** | 0.759 |
| **Extra-amygdala** | **CPu** | [2, 13]=3.16 | 0.076 | - | - | - |
|  | **Cg** | [2, 10]=0.22 | 0.807 | - | - | - |
|  | **PrL** | [2, 13]=0.41 | 0.672 | - | - | - |
|  | **IL** | [2, 10]=0.73 | 0.506 | - | - | - |
|  | **M1** | [2, 10]=0.17 | 0.844 | - | - | - |
|  | **BNST** | [2, 12]=0.26 | 0.774 | - | - | - |
|  | **SNR** | [2, 13]=0.48 | 0.627 | - | - | - |

**Table S3. Effects of stressin I and D1 antagonist SCH23390 on D1 receptor binding in amygdala regions.** [^3^H]-SCH23390 binding 1 hour after intra-amygdala injection of stressin I (0.01µg/0.5µl injection volume), stressin I + 120ng SCH23390 (0.01µg+120ng/0.5µl injection volume) and CSF (5µl injection volume) in rats. D1 binding data are shown as means±SEM. IN, intercalated amygdaloid nucleus, main part; Ivp, ventral paracapsular island; Imp, medial paracapsular intercalated cells; Idp, dorsal paracapsular intercalated cells; Ilp, lateral paracapsular intercalated cells; BLA, basolateral amygdala; CeA, central amygdala; BMA, basomedial amygdala; MeA, medial amygdala.

| **Region** | **CSF** | | **stressin I** | | **stressin I + SCH23390** | |
| --- | --- | --- | --- | --- | --- | --- |
|  | **Mean ± SEM** | **N** | **Mean ± SEM** | **N** | **Mean ± SEM** | **N** |
|  | **[fmol/mg]** |  | **[fmol/mg]** |  | **[fmol/mg]** |  |
| **IN** | 464.52 ± 11.80 | 5 | 547.62 ± 14.54 | 5 | 431.21 ± 17.90 | 5 |
| **Ivp** | 373.82 ± 7.85 | 5 | 464.27 ± 19.17 | 5 | 343.41 ± 7.56 | 5 |
| **Idp** | 356.67 ± 12.13 | 5 | 404.38 ± 11.47 | 4 | 353.08 ± 15.34 | 5 |
| **Imp** | 347.64 ± 10.44 | 5 | 428.65 ± 13.33 | 5 | 342.78 ± 7.78 | 5 |
| **Ilp** | 349.87 ± 13.14 | 5 | 447.81 ± 11.59 | 4 | 335.28 ± 6.20 | 5 |
| **BLA** | 243.13 ± 9.74 | 5 | 287.78 ± 8.70 | 4 | 248.96 ± 4.06 | 4 |
| **BMA** | 127.01 ± 6.81 | 4 | 202.40 ± 7.87 | 5 | 125.94 ± 2.99 | 5 |
| **CeA** | 66.28 ± 2.22 | 5 | 94.84 ± 6.45 | 5 | 64.84 ± 2.75 | 5 |
| **MeA** | 84.20 ± 5.68 | 5 | 111.10 ± 6.48 | 5 | 83.17 ± 2.87 | 5 |

Table S4. Regions with statistically significant effects on D1 receptor binding after intra-amygdala injection of stressin I and D1 antagonist SCH23390. [^3^H]-SCH23390 binding 1 hour after intra-amygdala injection of stressin I (0.01µg/0.5µl injection volume), stressin I + 120ng SCH23390 (0.01µg+120ng/0.5µl injection volume) and CSF (5µl injection volume) in rats. IN, intercalated amygdaloid nucleus, main part; Ivp, ventral paracapsular island; Imp, medial paracapsular intercalated cells; Idp, dorsal paracapsular intercalated cells; Ilp, lateral paracapsular intercalated cells; BLA, basolateral amygdala; CeA, central amygdala; BMA, basomedial amygdala; MeA, medial amygdala.

| **Region** | **F-value** | **p-value** | **CSF vs stressin I** | **CSF vs stressin I + SCH23390** | **stressin I vs stressin I + SCH23390** |
| --- | --- | --- | --- | --- | --- |
| **IN** | [2, 12]=16.07 | 0.0004 | 0.0020** | 0.141 | 0.0001*** |
| **Ivp** | [2, 12]=24.37 | 0.00006 | 0.0003*** | 0.117 | 0.00002*** |
| **Idp** | [2, 11]=4.19 | 0.0443 | 0.032* | 0.848 | 0.023* |
| **Imp** | [2, 12]=20.10 | 0.0001 | 0.0002*** | 0.755 | 0.0001*** |
| **Ilp** | [2, 11]=30.35 | 0.00003 | 0.00006*** | 0.338 | 0.00002*** |
| **BLA** | [2, 10]=8.28 | 0.0076 | 0.003** | 0.626 | 0.010* |
| **BMA** | [2, 11]=51.80 | 0.000003 | 0.000004*** | 0.907 | 0.000002*** |
| **CeA** | [2, 12]=15.88 | 0.0004 | 0.0005*** | 0.815 | 0.0003*** |
| **MeA** | [2, 12]=9.13 | 0.0039 | 0.0035** | 0.891 | 0.0027** |

Table S5. Effects of stressin I on D1 receptor binding in D1^Cre^-Crhr1^-/-^ knockout mice. [^3^H]-SCH23390 binding 1 hour after i.c.v. injection of stressin I (2µg/2µl injection volume) and CSF (2µl injection volume) in D1^Cre^-Crhr1^-/-^ mice and their Crhr1^f/f^ littermates. D1 binding data are shown as means±SEM. IN, intercalated amygdaloid nucleus, main part; Ivp, ventral paracapsular island; Imp, medial paracapsular intercalated cells; Idp, dorsal paracapsular intercalated cells; Ilp, lateral paracapsular intercalated cells; BLA, basolateral amygdala; CeA, central amygdala; BMA, basomedial amygdala; MeA, medial amygdala; CPu, caudate putamen; Cg, cingulate cortex; PrL, prelimbic cortex; IL, infralimbic region; M1, primary motor cortex; AcbC, nucleus accumbens core; AcbS, nucleus accumbens shell; BNST, bed nucleus of the stria terminalis; VTA, ventral tegmental area; SN, substantia nigra.

| **Region** | | **CRHR1^f/f^** | | | | **D1^Cre^-*Crhr1*^-/-^** | | | |
| --- | --- | --- | --- | --- | --- | --- | --- | --- | --- |
|  |  | **CSF** | | **stressin I** | | **CSF** | | **stressin I** | |
|  |  | **Mean**  **± SEM**  **[fmol/mg]** | **N** | **Mean**  **± SEM**  **[fmol/mg]** | **N** | **Mean**  **± SEM**  **[fmol/mg]** | **N** | **Mean**  **± SEM**  **[fmol/mg]** | **N** |
| **Amygdala** | **IN** | 936.33  ± 25.15 | 6 | 1116.55  ± 29.76 | 5 | 1046.02  ± 36.41 | 6 | 1057.75  ± 22.88 | 7 |
|  | **Imp** | 1014.58  ± 11.26 | 6 | 1151.32  ± 25.92 | 5 | 1072.55  ± 30.06 | 6 | 1094.37  ± 13.27 | 7 |
|  | **Ilp** | 737.15  ± 6.84 | 6 | 935.14  ± 31.58 | 4 | 801.72  ± 11.78 | 7 | 827.87  ± 14.37 | 7 |
|  | **BLA** | 349.34  ± 21.27 | 6 | 407.99  ± 11.71 | 5 | 378.56  ± 11.59 | 7 | 387.12  ± 8.79 | 7 |
|  | **CeA** | 212.35  ± 7.39 | 6 | 243.29  ± 7.19 | 5 | 221.41  ± 7.66 | 7 | 216.09  ± 9.03 | 6 |
|  | **BMA** | 251.41  ± 10.79 | 6 | 346.17  ± 3.91 | 5 | 326.20  ± 9.68 | 7 | 307.07  ± 7.07 | 7 |
|  | **MeA** | 227.80  ± 6.80 | 6 | 285.42  ± 6.36 | 5 | 244.92  ± 11.50 | 5 | 260.28  ± 7.04 | 7 |
| **Extra-amygdala** | **CPu** | 3591.54  ± 46.45 | 6 | 3697.13  ± 16.81 | 5 | 3637.43  ± 77.55 | 7 | 3640.86  ± 93.45 | 7 |
|  | **Cg** | 337.49  ± 23.27 | 6 | 312.52  ± 6.44 | 5 | 342.57  ± 16.44 | 7 | 315.83  ± 12.22 | 7 |
|  | **PrL** | 371.39  ± 11.84 | 6 | 364.14  ± 12.14 | 5 | 370.61  ± 14.97 | 7 | 347.70  ± 14.36 | 7 |
|  | **IL** | 290.44  ± 9.87 | 6 | 280.93  ± 9.48 | 5 | 293.80  ± 7.49 | 7 | 266.59  ± 10.09 | 7 |
|  | **M1** | 204.30  ± 16.82 | 6 | 174.26  ± 5.41 | 5 | 205.83  ± 15.39 | 7 | 189.15  ± 10.81 | 7 |
|  | **AcbC** | 2430.23  ± 89.04 | 6 | 2411.14  ± 134.64 | 5 | 2396.03  ± 80.04 | 4 | 2421.70  ± 94.12 | 6 |
|  | **AcbS** | 2722.65  ± 71.92 | 5 | 2602.43  ± 257.77 | 4 | 2568.43  ± 56.97 | 5 | 2651.82  ± 110.78 | 6 |
|  | **BNST** | 286.57  ± 6.60 | 6 | 293.60  ± 11.04 | 5 | 282.52  ± 5.42 | 7 | 285.26  ± 12.12 | 7 |
|  | **VTA** | 118.06  ± 11.13 | 6 | 124.46  ± 6.77 | 4 | 128.31  ± 8.41 | 6 | 137.12  ± 8.75 | 5 |
|  | **SN** | 1029.78  ± 13.03 | 6 | 1062.26  ± 41.76 | 5 | 1010.05  ± 9.48 | 6 | 1007.45  ± 18.97 | 5 |

Table S6. Regions with statistically significant effects on D1 receptor binding after i.c.v. injection of stressin I in D1^Cre^-Crhr1^-/-^ knockout mice. [^3^H]-SCH23390 binding 1 hour after i.c.v. injection of stressin I (2µg/2µl injection volume) and CSF (2µl injection volume) in D1^Cre^-Crhr1^-/-^ mice and their Crhr1^f/f^ littermates. IN, intercalated amygdaloid nucleus, main part; Ivp, ventral paracapsular island; Imp, medial paracapsular intercalated cells; Idp, dorsal paracapsular intercalated cells; Ilp, lateral paracapsular intercalated cells; BLA, basolateral amygdala; CeA, central amygdala; BMA, basomedial amygdala; MeA, medial amygdala; CPu, caudate putamen; Cg, cingulate cortex; PrL, prelimbic cortex; IL, infralimbic region; M1, primary motor cortex; AcbC, nucleus accumbens core; AcbS, nucleus accumbens shell; BNST, bed nucleus of the stria terminalis; VTA, ventral tegmental area; SN, substantia nigra.

| **Region** | | **Genotype effect** | **Treatment effect** | **Genotype x Treatment effect** |
| --- | --- | --- | --- | --- |
| **Amygdala** | **IN** | F[1, 20]=0.778  p=0.388 | F[1, 20]=11.069  p=0.003** | F[1, 20]=8.528  p=0.008** |
|  | **Imp** | F[1, 20]=0.000  p=0.981 | F[1, 20]=14.370  p=0.001** | F[1, 20]=7.550  p=0.012* |
|  | **Ilp** | F[1, 20]=1.860  p=0.187 | F[1, 20]=51.320  p=0.000001*** | F[1, 20]=30.170  p=0.00002*** |
|  | **BLA** | F[1, 21]=0.089  p=0.768 | F[1, 21]=5.769  p=0.026* | F[1, 21]=3.203  p=0.088 |
|  | **CeA** | F[1, 20]=1.284  p=0.271 | F[1, 20]=2.560  p=0.125 | F[1, 20]=5.130  p=0.035* |
|  | **BMA** | F[1, 21]=4.173  p=0.054 | F[1, 21]=18.737  p=0.0003*** | F[1, 21]=42.482  p=0.000002*** |
|  | **MeA** | F[1, 19]=0.248  p=0.624 | F[1, 19]=20.588  p=0.0002*** | F[1, 19]=6.905  p=0.017* |
| **Extra-amygdala** | **CPu** | F[1, 21]=0.005  p=0.944 | F[1, 21]=0.555  p=0.465 | F[1, 21]=0.487  p=0.493 |
|  | **Cg** | F[1, 21]=0.065  p=0.801 | F[1, 21]=2.480  p=0.130 | F[1, 21]=0.003  p=0.957 |
|  | **PrL** | F[1, 21]=0.378  p=0.545 | F[1, 21]=1.160  p=0.294 | F[1, 21]=0.313  p=0.582 |
|  | **IL** | F[1, 21]=0.342  p=0.565 | F[1, 21]=3.824  p=0.064 | F[1, 21]=0.888  p=0.357 |
|  | **M1** | F[1, 21]=0.363  p=0.553 | F[1, 21]=2.936  p=0.101 | F[1, 21]=0.240  p=0.629 |
|  | **AcbC** | F[1, 17]=0.013  p=0.911 | F[1, 17]=0.001  p=0.975 | F[1, 17]=0.046  p=0.833 |
|  | **AcbS** | F[1, 16]=0.161  p=0.693 | F[1, 16]=0.020  p=0.889 | F[1, 16]=0.609  p=0.447 |
|  | **BNST** | F[1, 21]=0.443  p=0.513 | F[1, 21]=0.275  P=0.605 | F[1, 21]=0.053  p=0.820 |
|  | **VTA** | F[1, 17]=1.439  p=0.247 | F[1, 17]=0.633  p=0.437 | F[1, 17]=0.016  p=0.901 |
|  | **SN** | F[1, 18]=2.734  p=0.116 | F[1, 18]=0.439  p=0.516 | F[1, 18]=0.605  p=0.447 |

**Table S7. D1 receptor binding in amygdala regions of 3-week abstinent rats and controls.** [^3^H]-SCH23390 binding in the amygdala of alcohol-dependent rats and controls. D1 binding data are shown as means ± SEM. IN, intercalated amygdaloid nucleus, main part; Ivp, ventral paracapsular island; Imp, medial paracapsular intercalated cells; Idp, dorsal paracapsular intercalated cells; Ilp, lateral paracapsular intercalated cells; BLA, basolateral amygdala; CeA, central amygdala.

| **Region** | **Control** | | **Alcohol dependent** | | **F-value** | **P-value** | **H-B corr. P-value** |
| --- | --- | --- | --- | --- | --- | --- | --- |
|  | **Mean ± SEM**  **[fmol/mg]** | **N** | **Mean ± SEM**  **[fmol/mg]** | **N** |  |  |  |
| **IN** | 802.48 ± 18.31 | 7 | 668.96 ± 17.18 | 8 | [1,13]=28.264 | 0.0001*** | 0.0008*** |
| **Ivp** | 1450.23 ± 115.09 | 6 | 731.26 ± 45.76 | 6 | [1,10]=33.699 | 0.0002*** | 0.0009*** |
| **Idp** | 859.38 ± 54.85 | 8 | 827.11 ± 39.16 | 7 | [1,13]=0.217 | 0.649 | 0.649 |
| **Imp** | 856.27 ± 63.18 | 7 | 667.50 ± 35.65 | 8 | [1,13]=7.242 | 0.019* | 0.056 |
| **Ilp** | 829.84 ± 30.67 | 7 | 720.29 ± 37.36 | 7 | [1,12]=5.137 | 0.043* | 0.085 |
| **BLA** | 512.04 ± 8.48 | 7 | 431.63 ± 10.92 | 6 | [1,11]=34.816 | 0.0001*** | 0.0007*** |
| **CeA** | 236.68 ± 7.46 | 8 | 179.71 ± 8.95 | 7 | [1,13]=24.299 | 0.0003*** | 0.001** |

**Table S8. *Drd1* mRNA expression in the amygdala of 3-week abstinent rats and controls.** Drd1a mRNA data are shown as means±SEM. IN, intercalated amygdaloid nucleus, main part; Ivp, ventral paracapsular island; Imp, medial paracapsular intercalated cells; Idp, dorsal paracapsular intercalated cells; Ilp, lateral paracapsular intercalated cells; BLA, basolateral amygdala; CeA, central amygdala.

| **Region** | **Control** | | **Alcohol dependent** | | **F-value** | **P-value** | **H-B corr. P-value** |
| --- | --- | --- | --- | --- | --- | --- | --- |
|  | **Mean ± SEM**  **[nCi/g]** | **N** | **Mean ± SEM**  **[nCi/g]** | **N** |  |  |  |
| **IN** | 83.89 ± 5.70 | 8 | 52.04 ± 6.69 | 7 | [1,13]=13.291 | 0.003** | 0.021* |
| **Ivp** | 83.78 ± 8.50 | 7 | 47.04 ± 9.05 | 6 | [1,11]=8.734 | 0.013* | 0.026* |
| **Idp** | 83.48 ± 4.78 | 7 | 55.95 ± 5.93 | 5 | [1,10]=13.283 | 0.005** | 0.023* |
| **Imp** | 48.85 ± 3.72 | 7 | 34.06 ± 1.02 | 4 | [1,9]=8.439 | 0.017* | 0.026* |
| **Ilp** | 64.98 ± 5.59 | 6 | 35.63 ± 4.63 | 5 | [1,9]=15.477 | 0.003** | 0.021* |
| **BLA** | 21.23 ± 1.55 | 8 | 14.18 ± 1.59 | 8 | [1,14]=10.099 | 0.007** | 0.023* |
| **CeA** | 6.61 ± 0.43 | 6 | 3.50 ± 0.79 | 5 | [1,9]=13.053 | 0.006** | 0.023* |

Table S9. No differences in spine density or spine length within the amygdala of alcohol dependent and control rats. Data are expressed as mean ± SEM values. For each group, five brains (n=5) were investigated. In each case, between 18 and 26 individual dendrites were mapped per region and brain. In total, more than 10000 individual dendritic spines were reconstructed per brain area (18815 in the CEA, 12431 in the MEA and 17733 in the BLA). CeA, central amygdala; MeA, medial amygdala; BLA, basolateral amygdala.

| Region | Measurements | Control rats  **Mean ± SEM** | Dependent rats  **Mean ± SEM** |
| --- | --- | --- | --- |
| CeA | Spine density per μm | 1.45 ± 0.04 | 1.51 ± 0.03 |
|  | Spine length in μm | 1.21 ± 0.04 | 1.18 ± 0.07 |
| MeA | Spine density per μm | 1.07 ± 0.02 | 1.03 ± 0.03 |
|  | Spine length in μm | 1.26 ± 0.04 | 1.29± 0.04 |
| BLA | Spine density per μm | 1.45 ± 0.02 | 1.52 ± 0.03 |
|  | Spine length in μm | 1.22 ± 0.02 | 1.19 ± 0.02 |

Table S10. Alcohol consumption in D1^Cre^-CRHR1^-/-^ knockout mice. Voluntary alcohol consumption (g/kg/day) in CRHR1^f/f^ and D1^Cre^-CRHR1^-/-^ mice three days after ethanol exposure (CIE) and air controls compared to baseline drinking before exposure. Alcohol intake is expressed in g/kg/day, mean ± SEM values. For statistical analysis the average of the 3 days of drinking after baseline was used. (*) Indicates the difference vs the baseline; (#) refers to the difference between the genotypes based on Fisher LSD post-hoc test.

|  | Control | | Dependent rats | |
| --- | --- | --- | --- | --- |
|  | CRHR1^f/f^  (N=12) | D1^Cre^-CRHR1^-/-^  (N=7) | CRHR1^f/f^  (N=6) | D1^Cre^-CRHR1^-/-^  (N=7) |
| Baseline | 4.88 ± 0.56 | 5.17 ± 0.35 | 5.14 ± 0.24 | 5.71 ± 0.61 |
| Day 1 | 11.95 ± 0.42 | 12.66 ± 0.62 | 19.45 ± 1.54 | 15.95 ± 0.81 |
| Day 2 | 10.97 ± 0.82 | 9.92 ± 1.79 | 19.02 ± 1.24 | 15.02 ± 1.16 |
| Day 3 | 8.38 ± 0.66 | 8.15 ± 1.36 | 17.66 ± 1.52 | 13.77 ± 0.71 |
| Average | 10.43 ± 0.55*** | 10.24 ± 0.93*** | 18.71 ± 1.32*** | 14.91 ± 0.66*** ^###^ |
