# Supplementary material for: Reducing stress and alcohol-related behaviors by targeting D1-CRHR1 receptor interactions in the amygdala
Source: Front Pharmacol. 2025 Oct 16;16:1677510. doi: 10.3389/fphar.2025.1677510 (PMC12571840; doi:10.3389/fphar.2025.1677510)
Supplement: Supplementary file 2 [file Image1.pdf]

SUPPLEMENTARY FIGURE S1

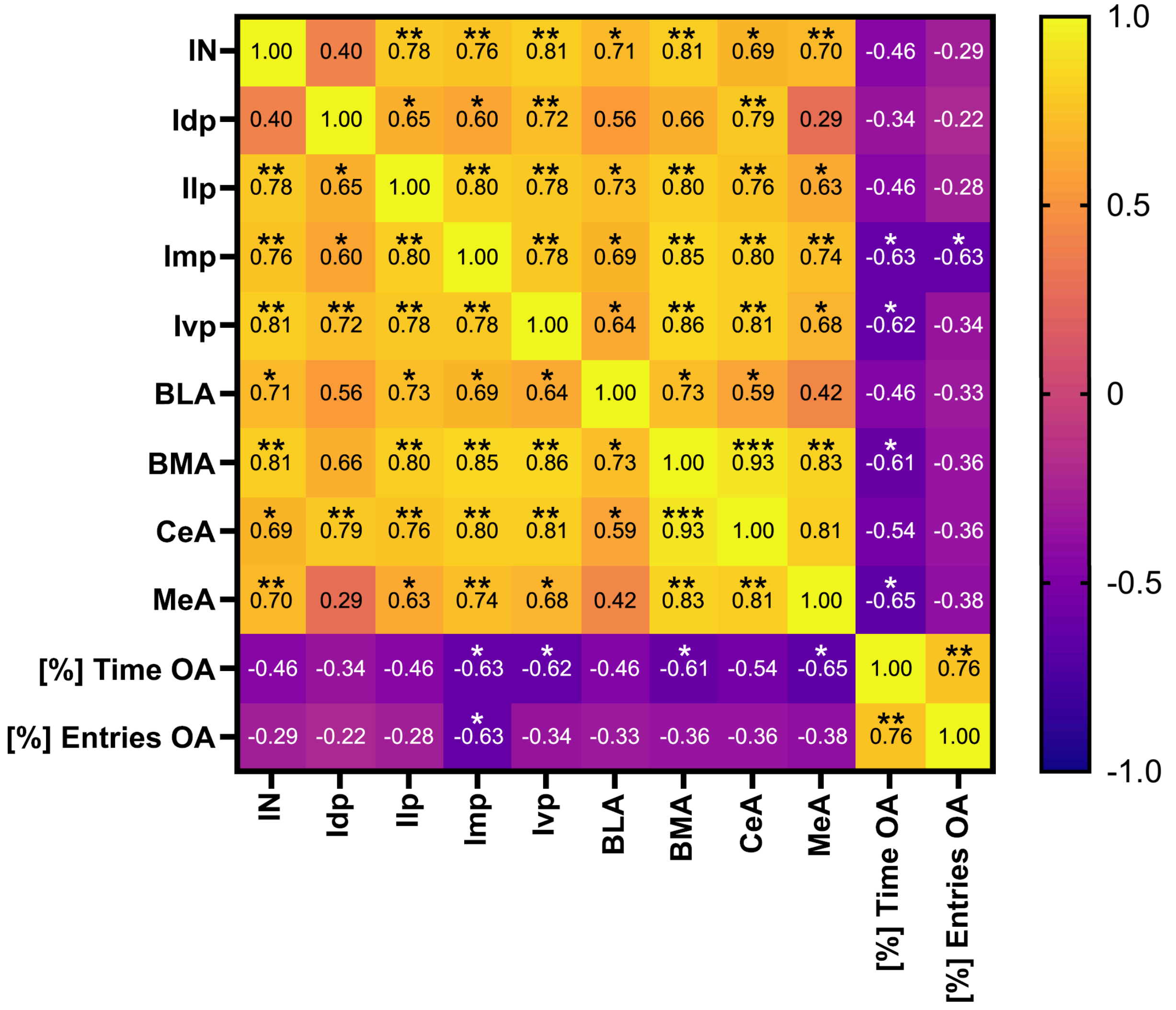

Supplementary Figure S1. Correlation matrix between D1 receptor expression in amygdala nuclei and intercalated clusters (ITCs) with behavioral outcomes in the elevated plus maze (percent time spent in the open arm and percent entries in the open arm). Pearson's correlation coefficients are shown as a color-coded heatmap, with yellow indicating strong positive correlations and violet indicating strong negative correlations. Significance levels are indicated (*$p < 0.05$, **$p < 0.01$, ***$p < 0.001$), and p values were corrected for multiple comparisons using the Benjamini–Hochberg false discovery rate (FDR) procedure. Data are from the same cohort of animals used in Figure 1F, in which rats received intra-amygdala injections of CSF vehicle, stressin I, or stressin I + SCH23390. IN: intercalated amygdaloid nucleus, main part; Idp: dorsal paracapsular intercalated cells; IIp: lateral paracapsular intercalated cells; Imp: medial paracapsular intercalated cells; Ivp: ventral paracapsular island; BLA: basolateral amygdala; BMA: basomedial amygdala; CeA: central amygdala; MeA: medial amygdala.
